# Supplementary material for: Beyond Templates and BERT: Headword-centric parsing for semantic question answering in non-english financial domains
Source: PLoS One. 2026 May 4;21(5):e0347261. doi: 10.1371/journal.pone.0347261 (PMC13138744; doi:10.1371/journal.pone.0347261)
Supplement: Attachment — Submitted filename: comments.pdf [file pone.0347261.s004.pdf]

The study focuses on improving **semantic question-answering (QA) systems** for **German financial texts**. Specifically, it introduces **INAGQA**, a new QA system designed to handle linguistic variability, headword-based parsing, domain-specific ambiguity resolution, and integration with knowledge graphs. The work evaluates this system on expert-annotated German financial queries and compares it against existing QA baselines like BERT-KGQA. The authors validate their approach on 2,100 expert-annotated German financial questions, reporting an F1 score of 0.91, outperforming both BERT-KGQA and template-based baselines while effectively handling temporal and quantitative question variants.

The logic and analysis of the paper are well organized. Therefore, I believe that this article has the potential to make a significant contribution to the field, provided that the major revisions outlined in the following comments are thoroughly addressed.

1. The methodology is said to rely on three theoretical pillars—Syntactic Dependency Theory, Knowledge Graph Embedding, and Human–AI Collaboration. It would be helpful if the authors could clarify how these pillars interact within the operational pipeline. While the components are described individually, the theoretical integration between them is not fully explained.

2. The system depends on predefined grammar rules in the NLTK chunker. The study would benefit from providing details on the coverage of these rules and how frequently the parser fails due to syntactic structures that fall outside the defined grammar. Quantifying the practical impact would strengthen the discussion.

3. Entity recognition is restricted to company names extracted from DBPedia. It would be valuable if the authors could explain how the system handles emerging companies or entities not included in this predefined list, especially in financial domains where new firms frequently appear.

4. Queries with multiple headwords are challenging, as noted in the study, and comparisons are made to EARL and Falcon. The paper would benefit from clarifying how INAGQA explicitly resolves conflicts arising from multiple extracted headwords.

5. The spelling-correction component interacts with complex or multi-word financial terms (e.g., Eigenkapitalrendite). It would be helpful if the authors could discuss how over-correction is avoided to prevent distortion of domain-specific terminology.

6. The Elasticsearch-based semantic search ranks IRIs based on similarity to headwords. The study would benefit from clarifying which similarity metric is used and how sensitive the ranking is to noisy, ambiguous, or partially extracted headwords.

7. System outputs include maps, info cards, or tables. It would be useful if the authors could explain the criteria or decision rules that determine which output format is selected for a given query.

8. Some newest papers are valued to refer, such as:

    1. https://doi.org/10.3390/app12199659
    2. https://doi.org/10.3390/app13031913
    3. https://doi.org/10.61186/jist.48127.13.49.24 (Alternative link: https://jist.ir/en/Article/48127)
