# Supplementary material for: Beyond Templates and BERT: Headword-centric parsing for semantic question answering in non-english financial domains
Source: PLoS One. 2026 May 4;21(5):e0347261. doi: 10.1371/journal.pone.0347261 (PMC13138744; doi:10.1371/journal.pone.0347261)
Supplement: Attachment — Submitted filename: Archivo PDF.pdf [file pone.0347261.s006.pdf]

PLOS One

Beyond Templates and BERT: Headword-Centric Parsing for Semantic Question Answering in Non-English Financial Domains

--Manuscript Draft--

**Manuscript Number:**	PONE-D-25-50479R1
**Article Type:**	Research Article
**Full Title:**	Beyond Templates and BERT: Headword-Centric Parsing for Semantic Question Answering in Non-English Financial Domains
**Short Title:**	Question Answering in Non-English Financial Domains
**Corresponding Author:**	Bassam Alshargabi<br>Cardiff Metropolitan University<br>cardiff, UNITED KINGDOM OF GREAT BRITAIN AND NORTHERN IRELAND
**Keywords:**	Semantic QA; headword parsing; Information retrieval; Entity/Relation; Linking; German NLP
**Abstract:**	Recent advances in semantic question-answering (QA) systems struggle with linguistic variability, particularly in non-English domains like German finance. This work presents INAGQA, a novel QA system that addresses this gap through headword-centric parsing, combining syntactic chunking with knowledge graph embeddings to resolve the question ambiguity. The main innovations are as follows: First, a hybrid disambiguation algorithm that achieves 0.91 F1 in German financial queries, validated on 2,100 expert-annotated questions. Second, domain-optimized shallow parsing with customizable grammar rules that reduces relation-linking errors by 35% for compound nouns (e.g., Eigenkapitalrendite). And finally, seamless knowledge integration to prioritize user-curated data and demonstrates 2.1s average response time in a case study with financial analysts. Our experiments show that INAGQA outperforms BERT-KGQA (F1: 0.83) and template-based systems (F1: 0.79) while handling temporal / quantitative variants (e.g., When vs. Where was X founded?) with 98% accuracy. The editable outputs of the system align with the Corporate Smart Insights frameworks, offering practical value for SMEs. To this end, the work contributes to IS research by proposing headword extraction as a replicable IS artifact for non-English QA and demonstrating language-sensitive design principles applicable to healthcare/legal domains
**Order of Authors:**	Jamal AlQundus
	Bassam Al-shargabi
**Opposed Reviewers:**	
**Response to Reviewers:**	Journal: PLOS ONE<br>Title: Beyond Templates and BERT: Headword-Centric Parsing for Semantic Question Answering in Non-English Financial Domains<br>Manuscript Number: PONE-D-25-50479<br>Date: 03 Feb 2026<br><br>Professor Mario Graff-Guerrero<br>Academic Editor, PLOS One<br>Dear Sir,<br>We sincerely thank you for the painstaking review undertaken for this paper. Thank you very much for your positive comments. They were gratifying. The constructive feedback has led to further improving the quality of the manuscript. Accordingly, we have modified the manuscript taking into consideration all the changes suggested by you.<br><br>We take it as a positive gesture that you have submitted progressive remarks about the manuscript. We have addressed the checklist.

Powered by Editorial Manager® and ProduXion Manager® from Aries Systems Corporation

Journal requirements:
Comment 1: “Please ensure that your manuscript meets PLOS ONE's style requirements, including those for file naming.”
Response: We further updated the manuscript to meet PLOS ONE's style requirements. The manuscript now uses the provided PLOS LaTeX template.

Comment 2: “In your Methods section, please include additional information about your dataset and ensure that you have included a statement specifying whether the collection and analysis method complied with the terms and conditions for the source of the data.”
Response: We have added a detailed description of the dataset, including source, collection method, preprocessing steps, and compliance with source terms. See updated Section Result and its Subsection Datasets. Part of the dataset used was manually annotated by domain experts and is considered part of the work contribution. That's why we decided to put it in Section Results, and not in Section Methodology.

Comment 3: “Please note that PLOS One has specific guidelines on code sharing for submissions in which author-generated code underpins the findings in the manuscript.”
Response: We will make the code publicly available on OneDrive upon acceptance. A link will be provided in the manuscript’s Data Availability Statement.

Comment 4: “Please update your submission to use the PLOS LaTeX template.”
Response: We are thankful to the editor for appreciating our work. We have further updated the manuscript and used the PLOS LaTeX template.

Comment 5: “Data availability”.
Response:
The Financial-QA dataset (2,100 German financial questions) cannot be shared publicly due to third-party licensing restrictions; however, for more informtion regarding the dataset can be made by contacting Silvio Peikert (Master of Science, Project Manager at Fraunhofer Institute for Open Communication Systems) at silvio.peikert@fokus.fraunhofer.de. The Virtuoso triple store contains proprietary financial announcements; however, a sample dataset (dbo2.json, dbo.json) is provided as Supporting Information

Reviewer 1
Comment 1: “The manuscript requires methodological clarifications: The methodology is said to rely on three theoretical pillars—Syntactic Dependency Theory, Knowledge Graph Embedding, and Human–AI Collaboration. It would be helpful if the authors could clarify how these pillars interact within the operational pipeline. While the components are described individually, the theoretical integration between them is not fully explained.”
Response: We have incorporated theoretical integration and added clarifications in Section Methodology regarding the interaction of theoretical pillars, rule coverage, entity recognition for new companies, multi-headword conflict resolution, and similarity metrics.

Comment 2: “The system depends on predefined grammar rules in the NLTK chunker. The study would benefit from providing details on the coverage of these rules and how frequently the parser fails due to syntactic structures that fall outside the defined grammar. Quantifying the practical impact would strengthen the discussion.”
Response: We have incorporated failure analysis in the last paragraph of Subsection Question Pre-processing

Comment 3: “Entity recognition is restricted to company names extracted from DBPedia. It would be valuable if the authors could explain how the system handles emerging companies or entities not included in this predefined list, especially in financial domains where new firms frequently appear.”
Response: We have incorporated handling emerging companies not yet in DBPedia in the last paragraph of Subsection Question Pre-processing

Comment 4: “Queries with multiple headwords are challenging, as noted in the study, and comparisons are made to EARL and Falcon. The paper would benefit from

Powered by Editorial Manager® and ProduXion Manager® from Aries Systems Corporation

clarifying how INAGQA explicitly resolves conflicts arising from multiple extracted headwords."
Response: We have incorporated details of dealing with multiple headwords at the end of Subsection Question Processing

Comment 5: "The spelling-correction component interacts with complex or multi-word financial terms (e.g., Eigenkapitalrendite). It would be helpful if the authors could discuss how over-correction is avoided to prevent distortion of domain-specific terminology."
Response: We have incorporated spelling correction details in Subsection Question Pre-processing

Comment 6: "The Elasticsearch-based semantic search ranks IRIs based on similarity to headwords. The study would benefit from clarifying which similarity metric is used and how sensitive the ranking is to noisy, ambiguous, or partially extracted headwords."
Response: We have incorporated details clarifying the used similarity metric in Subsection Workflow starting from "To determine the similarity between two phrases, the cosine similarity of their respective word vector is computed …"

Comment 7: "System outputs include maps, info cards, or tables. It would be useful if the authors could explain the criteria or decision rules that determine which output format is selected for a given query."
Response: We incorporated output format and selection criteria in Subsection Request Answer and Subsection Output and Curation

Comment 8: Some newest papers are valued to refer, such as:
1. https://doi.org/10.3390/app12199659
2. https://doi.org/10.3390/app13031913
3. https://doi.org/10.61186/jist.48127.13.49.24 (Alternative link: https://jist.ir/en/Article/48127)
Response: We added all suggested references in Section Related Work

Specific Points Addressed:
•Theoretical integration added at the beginning of Section Methodology.
•Grammar rule coverage detailed in Subsection Workflow
•Entity recognition for new companies addressed via Wikidata fallback (Subsection Suggestion).
•Multi-headword conflict resolution via similarity ranking (Subsection SPARQL Mapping).
•Spelling correction details added (Subsection Question Pre-processing).
•Similarity metric specified as cosine similarity with BERT embeddings (Subsection SPARQL Mapping).
•Output format selection criteria added in Subsections Request Answer and Output and Curation
•Suggested references have been reviewed and cited where relevant.

Reviewer 2
Comment 1: "However, the paper is lengthy and can be shorten as a concise version. For example, the workflow section is excessively detailed, including explanations of well-established concepts such as POS-tagging that are common knowledge in the NLP community. The paper would be more accessible if streamlined to focus on the novel contributions rather than providing textbook-level explanations of standard techniques."
Response: We thank the reviewer for the valuable suggestion. We have significantly streamlined the manuscript, particularly in the Methodology section, by removing basic NLP explanations (e.g., detailed descriptions of POS-tagging and syntactic parsing). The revised paper now focuses on our novel contributions: the headword-centric parsing algorithm, the hybrid rule- and embedding-based architecture, and the

Powered by Editorial Manager® and ProduXion Manager® from Aries Systems Corporation

system's practical integration.

Comment 2: “While the authors present a carefully designed workflow, the rationale behind key methodological choices is not well explained. For the first step, they use syntactic parsing to do pattern match, is it scalable or how is the coverage of this pattern detection? Why not any DL-model based solutions? In addition, in order to generate SPARQL query, they used Spacy’s standard NER, why not choose any other deep learning methods. Can you add a dedicated subsection justifying each major architectural choice with empirical evidence or theoretical reasoning?”
Response: We have added a new dedicated subsection in Section Methodology subsection Rationale for Architectural Choices. This subsection explicitly addresses the reviewer's questions:
Scalability and Coverage of Pattern Matching: We clarify that the system employs 46 handcrafted grammar rules, which achieved 92% coverage on our test set of 2,100 questions. Failure cases and error analysis are discussed in Subsection Question Pre-processing.
Choice of Rule-Based Parsing over Deep Learning (DL): We justify this choice based on: (1) Interpretability and control for decomposing German compound nouns, (2) Data efficiency, as large annotated German financial QA datasets are scarce, and (3) Lower operational latency and cost compared to querying large LLM APIs, which is crucial for enterprise deployment.
Choice of SpaCy NER: We explain that SpaCy's de_core_news_lg model offers robust, fast entity recognition for German. As our core financial entity list (companies) is stable, we augmented it with a custom EntityRuler for domain terms, achieving high precision without the need for training a custom DL model.

Comment 3: “The scalability and generalizability of the approach remain uncertain. The system relies on handwritten grammar rules for pattern matching, but the paper does not clarify: (1) how many rules were created for the 2,100 test questions, (2) what coverage can be expected for unseen question variations, and (3) how much expert effort is required to extend the system to new financial concepts or question types. This raises concerns about the practical scalability of the rule-based approach.”
Response: We have provided the requested clarifications:
Number of Rules: As stated above, 46 handcrafted grammar rules were developed (Subsection Rationale for Architectural Choices).
Coverage for Unseen Variations: These rules covered 92% of the 2,100 test questions. We discuss the nature of the 8% failure cases (primarily highly elliptical or metaphoric questions) and our fallback strategy using embedding-based similarity ranking in Subsection Question Pre-processing
Expert Effort for Extension: The modular design of the rule system allows incremental extension. Adding a new question pattern or financial concept typically requires 1-2 new rules, which can be authored by a domain expert in collaboration with a developer. This process and its manageability are discussed in Subsection Limitations and Future Work.

Comment 4: “With recent advances in LLMs, many sub-tasks can now be automated through in-context learning. However, the paper lacks comparison with current SOTA approaches such as GPT-4, Claude, or fine-tuned multilingual LLMs. It is more of a comparison of rule-based methods v.s. automated solutions.”
Response: We agree that a comparison with modern LLMs is essential. We have added a new part in Section Discussion Subsection Comparison with Large Language Models. This section
reports a comparative evaluation of GPT-4 (using few-shot prompting) on a subset of our Financial-QA dataset.
discusses the results: While GPT-4 performs well on factual queries, INAGQA maintains a significant advantage (15% higher F1-score) on ambiguous queries and complex German compound nouns.
provides a reasoned discussion on the trade-offs: LLMs offer generality but at higher cost, latency, and with less interpretability and control for specific, sensitive financial domains and highlights the continued relevance of our specialized, hybrid approach.

Reviewer 3
Comment I-III (Weaknesses): “Concerns regarding data availability, experimental setup clarity, baseline configuration, absence of ablation study and LLM comparison, and

	claims vs. evidence on cross-domain applicability."<br>Response to Weaknesses: We thank the reviewer for these critical points, which have significantly strengthened the manuscript.<br>Data Availability: We have the manuscript and added section Data Availability Statement in full compliance with PLOS ONE policy. The proprietary Financial-QA dataset (2,100 questions) will be made available upon request for research purposes. A sample dataset (dbo.json) is provided as Supporting Information.<br>Dataset details, including source, collection method, preprocessing, and annotation guidelines, are now provided in section Results subsection Datasets. We also report an inter-annotator agreement (IAA) score (Cohen's $\kappa = 0.85$) for the annotated questions.<br>Experimental Setup & Baselines: We clarify that all baseline systems (Falcon 2.0, EARL, BERT-KGQA) were retrained/fine-tuned on our German financial dataset to ensure a fair comparison. Training details (data splits, hyperparameters) are provided in section Results subsection Performance Comparison.<br>The specific embedding model used for similarity ranking in INAGQA is bert-base-german-cased, as now specified in section Methodology subsection SPARQL Mapping.<br>Ablation Study: We have added a new Section Results subsection Ablation Study. This study isolates the contribution of key components (pattern matching, headword extraction, similarity ranking), clearly demonstrating that the full hybrid pipeline delivers the best performance.<br>LLM Comparison: As detailed in the response to Reviewer #2 (Comment 4), we have added a comprehensive comparison with GPT-4 in Section Discussion Subsection Comparison with Large Language Models.<br>Cross-Domain Applicability: We have clarified the results in Table 6. The performance drop in healthcare/legal domains occurred when using financial-domain rules without modification. We note that adapting the system requires developing domain-specific rules, which is feasible given the modular architecture. This discussion on generalizability is now in Section Conclusion and Further Work Subsection Methodological Innovation.<br><br>Comment IV (Questions to Authors):<br>Q1. Dataset specification and experimental setup:<br>Datasets: Tables 4, 5, and 6 use our Financial-QA dataset. The Virtuoso triple store (14k announcements) was used for the case study in Section Results Subsection Datasets.<br>Train/Test Split: The dataset was split 80/20 (1,680 training / 420 test questions) for development and final evaluation. All reported results (including baselines) are on the held-out test set.<br>Q2. Baseline configuration: All baselines were fine-tuned for German using our training split. We used the authors' original code where available and adapted training pipelines for the German financial domain. Specifics are in Section Results Subsection Experimental Setup.<br>Embedding models: INAGQA uses bert-base-german-cased. BERT-KGQA used the same for fairness. Falcon and EARL used their standard multilingual embeddings, as per their design.<br>Q3. Cross-domain evaluation: Healthcare/legal test questions were sourced from public German QA benchmarks and curated with domain experts. For Table 6, we applied the Financial-QA rules without modification to test "out-of-the-box" performance, hence the noted drop. Successful adaptation, as discussed, requires adding domain-specific rules.<br><br>Comment V (Writing style and organizational suggestions):<br>Redundant Content: We have consolidated the manuscript, removing duplicate explanations of entity recognition and SPARQL generation (now primarily in Section Methodology).<br>Undefined Terms: We now define Corporate Smart Insights (our overarching analytics framework) and OMG API (the gateway for knowledge base queries) upon their first use in Section Introduction and Section Architecture.<br><br>Comment VI (Typographical errors):<br>Table 7 Legend: Added a clear legend: "✓ indicates correct answer, o indicates failure or incorrect answer."

	Incorrect Reference: Corrected the reference in Section Discussion from Table 2 to Table 7.<br><br>Reviewer 4<br>Comment 1.1 & 1.2 (Data Availability & Annotation):<br>Response: We have fully addressed these concerns.<br>The Data Availability Statement (Section Conclusion and Further Work) now clearly states the conditions for accessing the proprietary Financial-QA dataset and provides a sample (dbo.json).<br>In Section Results Subsection Datasets, we detail the annotation process. The 2,100 questions were annotated by two domain experts. We report an inter-annotator agreement (IAA) score of Cohen's $\kappa = 0.85$, indicating substantial agreement. Discrepancies were resolved through discussion.<br>Comment 2.1 & 2.2 (Evaluation - Baseline Fairness & Cross-Validation):<br>Response: We thank the reviewer for highlighting the need for rigorous evaluation.<br>Baseline Fairness: We confirm that all baseline systems (Falcon 2.0, EARL, BERT-KGQA) were fine-tuned on the same German financial training data (1,680 questions) as INAGQA. Hyperparameter tuning details and training convergence criteria are provided in Section Results Subsection Performance Comparision.<br>Cross-Validation: To provide a more robust performance estimate, we have conducted 5-fold cross-validation on the Financial-QA dataset. The results (average F1-score of 0.89 ± 0.02) are presented in a new Table 5. The headline F1-score of 0.91 in the abstract and results refers to the performance on the final, held-out test set, consistent with the cross-validation average.<br><br>CONCLUDING REMARKS<br>We thank the reviewers and editor for their valuable feedback, which has significantly improved the manuscript. Since all the changes that you requested have been incorporated, we sincerely hope that the manuscript is in its final publishable form.<br><br>With Gratitude,<br>The Authors
**Additional Information:**	
**Question**	**Response**
**Financial Disclosure**<br><br>Enter a financial disclosure statement that describes the sources of funding for the work included in this submission. Review the submission guidelines for detailed requirements. View published research articles from *PLOS ONE* for specific examples.<br><br>This statement is required for submission and **will appear in the published article** if the submission is accepted. Please make sure it is accurate.	The author(s) received no specific funding for this work.

**Funded studies**<br>Enter a statement with the following details:<br>• Initials of the authors who received each award<br>• Grant numbers awarded to each author<br>• The full name of each funder<br>• URL of each funder website<br>• Did the sponsors or funders play any role in the study design, data collection and analysis, decision to publish, or preparation of the manuscript?<br><br>Did you receive funding for this work?	
**Competing Interests**<br><br>Use the instructions below to enter a competing interest statement for this submission. On behalf of all authors, disclose any competing interests that could be perceived to bias this work—acknowledging all financial support and any other relevant financial or non-financial competing interests.<br><br>This statement is required for submission and **will appear in the published article** if the submission is accepted. Please make sure it is accurate and that any funding sources listed in your Funding Information later in the submission form are also declared in your Financial Disclosure statement.<br><br>View published research articles from *PLOS ONE* for specific examples.	The authors have declared that no competing interests exist.

Powered by Editorial Manager® and ProduXion Manager® from Aries Systems Corporation

**NO authors have competing interests**<br><br>Enter: *The authors have declared that no competing interests exist.*<br><br>**Authors with competing interests**<br><br>Enter competing interest details beginning with this statement:<br><br>*I have read the journal's policy and the authors of this manuscript have the following competing interests: [insert competing interests here]*<br><br>* typeset	
**Ethics Statement**<br><br>Enter an ethics statement for this submission. This statement is required if the study involved:<br><br>• Human participants<br>• Human specimens or tissue<br>• Vertebrate animals or cephalopods<br>• Vertebrate embryos or tissues<br>• Field research<br><br>Write "N/A" if the submission does not require an ethics statement.<br><br>General guidance is provided below. Consult the submission guidelines for detailed instructions. **Make sure that all information entered here is included in the Methods section of the manuscript.**	Not applicable

**Format for specific study types**<br><br>**Human Subject Research (involving human participants and/or tissue)**<br>• Give the name of the institutional review board or ethics committee that approved the study<br>• Include the approval number and/or a statement indicating approval of this research<br>• Indicate the form of consent obtained (written/oral) or the reason that consent was not obtained (e.g. the data were analyzed anonymously)<br><br>**Animal Research (involving vertebrate animals, embryos or tissues)**<br>• Provide the name of the Institutional Animal Care and Use Committee (IACUC) or other relevant ethics board that reviewed the study protocol, and indicate whether they approved this research or granted a formal waiver of ethical approval<br>• Include an approval number if one was obtained<br>• If the study involved *non-human primates*, add *additional details* about animal welfare and steps taken to ameliorate suffering<br>• If anesthesia, euthanasia, or any kind of animal sacrifice is part of the study, include briefly which substances and/or methods were applied<br><br>**Field Research**<br><br>Include the following details if this study involves the collection of plant, animal, or other materials from a natural setting:<br>• Field permit number<br>• Name of the institution or relevant body that granted permission	
**Data Availability**<br><br>Authors are required to make all data underlying the findings described fully available, without restriction, and from the time of publication. PLOS allows rare exceptions to address legal and ethical concerns. See the PLOS Data Policy and FAQ for detailed information.	No - some restrictions will apply

A Data Availability Statement describing where the data can be found is required at submission. Your answers to this question constitute the Data Availability Statement and **will be published in the article**, if accepted.<br><br>**Important:** Stating 'data available on request from the author' is not sufficient. If your data are only available upon request, select 'No' for the first question and explain your exceptional situation in the text box.<br><br>Do the authors confirm that all data underlying the findings described in their manuscript are fully available without restriction?	
**Describe where the data may be found in full sentences. If you are copying our sample text, replace any instances of XXX with the appropriate details.**<br><br>• If the data are **held or will be held in a public repository**, include URLs, accession numbers or DOIs. If this information will only be available after acceptance, indicate this by ticking the box below. For example: *All XXX files are available from the XXX database (accession number(s) XXX, XXX.).*<br>• If the data are all contained **within the manuscript and/or Supporting Information files**, enter the following: *All relevant data are within the manuscript and its Supporting Information files.*<br>• If neither of these applies but you are able to provide **details of access elsewhere**, with or without limitations, please do so. For example:<br><br>*Data cannot be shared publicly because of [XXX]. Data are available from the XXX Institutional Data Access / Ethics Committee (contact via XXX) for researchers who meet the criteria for access to confidential data.*<br><br>*The data underlying the results presented in the study are available from (include the name of the third party*	All data available within the paper

and contact information or URL).<br>• This text is appropriate if the data are owned by a third party and authors do not have permission to share the data.<br><br>* typeset	
Additional data availability information:	

Cover Letter

Dear Editors,

On behalf of my co-author, I am pleased to submit our manuscript entitled "Beyond Templates and BERT: Headword-Centric Parsing for Semantic Question Answering in Non-English Financial Domains" for consideration in PLOS ONE. This study introduces INAGQA, a novel semantic question-answering system that addresses a key challenge in natural language processing: linguistic variability in non-English, domain-specific contexts. By combining shallow syntactic parsing, headword extraction, and hybrid disambiguation with knowledge graph embeddings, our system achieves state-of-the-art performance in German financial question answering (F1 = 0.91) while maintaining computational efficiency and explainability. The contributions of this work are threefold:

- Methodological innovation : We propose a hybrid disambiguation algorithm that outperforms existing template-based and deep learning approaches, particularly in low-resource languages.
- Theoretical significance : We demonstrate that headword-centric parsing is a replicable artifact for information systems research and can be generalized to domains beyond finance, such as healthcare and legal contexts.
- Practical value : Through a case study with financial analysts, we validate the system's usability, showing fast response times (2.1s on average) and transparent outputs that support user curation and knowledge evolution.

This manuscript has not been published elsewhere and is not under consideration by another journal. All authors have approved the manuscript and agree with its submission to PLOS ONE.

We believe our study will be of broad interest to the PLOS ONE readership, as it advances natural language processing, knowledge graph integration, and intelligent information systems, with implications for both scientific research and practical applications in decision support.

Thank you for your time and consideration. We look forward to your response.

Sincerely,

Dr. Bassam Al-shargabi
Cardiff School of Technologies,

Cardiff Metropolitan University
Email: bal-shargabi@cardiffmet.ac.uk

Manuscript

Click here to access/download;Manuscript;PLOS__Revised_Manuscript_Clea

Beyond Templates and BERT: Headword-Centric Parsing for Semantic Question Answering in Non-English Financial Domains

Jamal Al Qundus[1]◑, Bassam Alshargabi[2*]◑ ¶

1 Business Intelligence and Data Analytics, German Jordanian University, Amman, Jordan
2 Cardiff School of Technologies, Cardiff Metropolitan University, Cardiff, UK

◑These authors contributed equally to this work.
¤Current Address: Cardiff School of Technologies, Cardiff Metropolitan University, Cardiff, UK
* bal-shargabi@cardiffmet.ac.uk

Abstract

Recent advances in semantic question-answering (QA) systems struggle with linguistic variability, particularly in non-English domains like German finance. This work presents INAGQA, a novel QA system that addresses this gap through headword-centric parsing, combining syntactic chunking with knowledge graph embeddings to resolve the question ambiguity. The main innovations are as follows: First, a hybrid disambiguation algorithm that achieves 0.91 F1 in German financial queries, validated on 2,100 expert-annotated questions. Second, domain-optimized shallow parsing with customizable grammar rules that reduces relation-linking errors by 35% for compound nouns (e.g., *Eigenkapitalrendite*). And finally, seamless knowledge integration to prioritize user-curated data and demonstrates 2.1s average response time in a case study with financial analysts. Our experiments show that INAGQA outperforms BERT-KGQA (F1: 0.83) and template-based systems (F1: 0.79) while handling temporal / quantitative variants (e.g., *When* vs. *Where was X founded?*) with 98% accuracy. The editable system's outputs of the system align with the Corporate Smart Insights frameworks, offering practical value for SMEs. To this end, the work contributes to IS research by proposing headword extraction as a replicable IS artifact for non-English QA and demonstrating language-sensitive design principles applicable to healthcare/legal domains.

Introduction

In recent years, approaches incorporate unstructured text into machine-readable representations, as well as the use of knowledge graphs [1] have become crucial assets for innumerable information extraction and knowledge discovery projects [2]. AI knowledge that has been semantically curated enables the effective application of knowledge artifacts. As a result, this makes a significant contribution to improving functional quality, data efficiency, plausibility verification, and the dependability of AI-based functions in a variety of information retrieval systems. The exponential growth of structured knowledge bases (e.g. Wikidata [3], DBpedia [4]) has intensified the demand for precise natural language question answering systems (QA) [5]. Although existing systems excel at factual queries (e.g., *What is Siemens' revenue?*), they struggle with

question ambiguity, where variants like *Where was Siemens founded?* vs. *When was Siemens founded?* require distinct relation linking [6]. This challenge is acute in financial domains, where terminology varies across languages (e.g., German compound nouns like *Eigenkapitalrendite*).

Existing Approach Categories

Current approaches fall into three categories: First, Template-based QA [7]: Handles fixed patterns, but fails on free-text questions. Second, Deep Learning QA [8]: Requires massive training data and struggles with low-resource languages. Finally, Hybrid Semantic Parsing [9]: Balances flexibility and precision, but lacks robust headword disambiguation.

This work bridges this gap with INAGQA. This German financial QA system that introduces a headword-centric parsing algorithm (section Methodology) combining shallow syntax trees with semantic similarity ranking, achieving 22% higher F1-score than Falcon 2.0 in ambiguous queries (section Results), proves domain adaptability through a case study on 14,000 German financial announcements, outperforms template-based systems by 35% on compound noun queries, and offers practical utility via integration with corporate knowledge graphs [10], enabling dynamic curation of financial insights.

Our work advances IS research by proposing headword extraction as a novel IS artifact for QA systems and demonstrating language-sensitive design principles for non-English domains [11]. It focuses on the task of knowledge-based question answering to address two major challenges. The vagueness of the question variants presents a difficulty in determining the question's intention , which is typically connected to a certain context that is not known by the system. Another difficulty is the variety of a question, for example *where is the company located? What is the company's address? Where is the main office of the company? Where can I find the company address? Where is the company? Where does the company occur?* To overcome the aforementioned challenges, many methods have restricted the potential questions within a particular domain that is based on methods such as predefined questions or a controlled vocabulary to generate questions, which make use of a knowledge graph (e.g. Wikidata [12], DBPedia [13], YAGO [14], KnowItAll [15]) for mapping to SPARQL-Queries [16].

Although this work is implemented on DBPedia as a knowledge base and rely on its structure to create the RDF triples store, the INAGQA system applies Natural Language Processing (NLP) and other techniques to investigate questions similar to AskNow [17], EARL [18], and FALCON [19]. Also, it includes Pattern Matching and Headwords algorithms for the purpose of supporting covering a greater range of question variants and being more elastic in handling free-text questions, which is a significant improvement of existing works (section Results).

The proposed hybrid semantic information retrieval system INAGQA is an extension of a previous research [20] as a question-answering system. It allows posing free-text questions on finance in German to target RDF knowledge bases: DBpedia, virtuoso (our own created triple-store), and Wikidata. INAGQA can classify questions with respect to their included intention by identifying the relevant entities and their semantic relation. Questions are processed through a processing pipeline of approaches i.e., Pattern-Matching, Headwords extraction, and extended Relation-Linking. Generated SPARQL-Queries are sent to an OMG API for Knowledge that covers functions such as spellchecking and a message broker with agents to monitor the responses and take actions if required.

Work Contributions

The contributions of this work can be summarized as : A novel pipeline of AI components for classifying questions in terms of intentions. This is stable and showed resistance to question variation. An extended approach including handwritten rules, quantifiers mapping, and similarity ranking can be used for entity- and relation-linking. A translation component that facilitates the conversion of natural language queries to SPARQL queries. This enables a more flexible utilization of the Semantic Web infrastructure, including triple stores, and supports the formulation of complex questions. The syntax trees are employed to identify headwords essential for relation-linking by providing syntactic dependency information. Lastly, the incorporation of advanced infrastructure for a question-answering system, including a spellchecker, and an agent system for data curation.

The paper is organized as follows. State-of-the-art related works are presented in Section . Section gives an overview of the architecture of the question-answering system. Section explores the provided architecture in more detail and describes the methodology. Section describes the results obtained, which are discussed in Section and then concluded with directions for future work in Section .

Related Work

This section gives a brief overview of related works that focus on approaches to developing question-answering systems and provides a summarized comparative analysis as in Table 1.

Question Analysis in QA Systems

Recent work in semantic parsing has revealed critical gaps in handling question variability (Mohamed et al. 2024). Template-based approaches [21] achieve 0.81 precision on fixed patterns but drop to 0.52 on free-form questions such as German compound queries. Neural methods [22] show promise, but require massive training data (10^5 annotated training examples), a limitation that our headword technique circumvents through rule-based chunking. Hybrid systems [23,24] combine entity-relation linking but struggle with temporal/quantitative ambiguity (e.g., *When* vs. *How much*), which our similarity ranking addresses.

Headword Extraction Techniques

The identification of Headword has evolved from the following.

- Dependency Parsing [25]: Achieves 0.88 recall, but is computationally expensive.
- Shallow Chunking [26]: Faster but less precise (0.68).
- Hybrid Approaches: Our work bridges this gap by:
  - Using Spacy's[1] efficient tokenization
  - Adding financial-domain grammar rules (section Methodology)
  - Achieving 0.91 F1-Score with lower resource demands (Table 4)

[1] https://spacy.io

Financial Domain Applications

Prior financial QA systems face three key limitations:

- Language bias: Most target English, even GPT-4o struggle in non-English achieves 42% accuracy [27]
- Static Templates: Cannot handle emerging terms like *ESG metrics* [28]
- Black-box Models: Lack of explainability for regulatory compliance [29]

Proposed Work

As illustrated in Table 1, INAGQA advances the field by:

- Incorporating dynamic rule updates via user curation (subsection)
- Providing auditable parsing logs for financial regulators
- Supporting German compound nouns through chunking rules $(NP \rightarrow DETADJ * NOUN)$

Table 1. shows a comparative analysis of the related work

Approach	Strength	Weakness	Our Improvement
Template-based	High precision (0.81)	Rigid patterns	Dynamic grammar rules
Neural QA	Handles variability	Data-hungry	Rule-based efficiency
Hybrid (Falcon)	Combined linking	Temporal ambiguity	Headword ranking

Review

Semantic parsing-based QA systems employ entity and relation-linking tasks to establish connections between key phrases, concepts, and their relationships in knowledge bases, which form the fundamental pipeline for measuring semantic similarity. Recent advances in contrastive learning for semantic similarity, such as SupMPN [30] and SelfCCL [31], demonstrate improved sentence embedding quality by discriminating between multiple positive and negative examples. These methods address BERT's anisotropy and could enhance relation-linking robustness in multilingual QA. Similarly, BERTurk-contrastive [32] shows promising results for low-resource languages, supporting our focus on German financial NLP. We explored the latest developments in this field, including AskNow, and examined their effectiveness in performing these tasks. Qsearch [33] and GQA [34] consider entity linking, and IQA [35], while KBPearl [36], EARL [18], and Falcon [37] consider both tasks. All of them use DBPedia Knowledge Graph (KG) for entity extraction.

AskNow [17] relies primarily on a rule-based information retrieval system approach, which converts natural language questions in free text into a formal query language. It utilizes DBPedia for entity linking and introduces a syntactic structure called Normalized Query Structure (NQS) to act as an intermediary canonical form for natural language questions before translating them to SPARQL-Queries. NQS utilizes part-of-speech (PoS) tagging to identify query desires such as noun phrases, which is similar to our approach in extracting headwords from the given question. However, AskNow's reliance on language models and complex development processes makes it difficult to reproduce. Qsearch [33] as a question-answering system focuses on handling quantity conditions present in the question text. The system uses a rule-based parser to

map input questions to Qqueries, which are then processed by a deep neural network to extract quantity-centric facts called Qfacts. These facts are assigned a score based on their relevance to the mapped Qquery. This work is relevant in exploring the study of quantity conditions. In contrast to QSearch, GQA [34] is a Grammatical Question Answering system that prioritizes grammar over quantity conditions. It facilitates English language queries over DBPedia using a set of conceptual grammar derived from the Grammatical Framework (GF) to parse questions with complex syntactic constructions. Similarly, the proposed INAGQA system involves creating syntactic trees for German to classify user input questions based on their intent and expected answer type through interaction with the user using IQA [35] and incorporates user feedback into the question-answering process using an interaction scheme for a semantic Question-Answering pipeline (PL). After the entity recognition phase, the PL generates a set of entity-related questions that the user can answer to interpret the original question to present a metric called Option Gain to efficiently integrate user feedback through interaction.

KBPearl [36] aims to tackle the problem of knowledge base population (KBP) by forming knowledge bases from unstructured text by combining the relation linking with joint entity. The authors have acknowledged the problem of incongruence caused by duplication and uncertainty when producing KBP information using comparable methodologies. The technique begins with an insufficient knowledge base and a set of texts and utilizes Open Information Extraction (Open IE) to fragment the texts into facts and side information, which constitutes metadata taken from the original text. However, the use of this approach is restricted to specific categories of data since some source materials may not contain such metadata, such as time stamps. EARL [18] constructs a series of procedures for entity linking and relation linking by integrating both tasks and proposes two approaches to solve them. Firstly, it formulates the tasks as an example of the Generalized Travelling Salesman Problem (GTSP), a problem known to be NP-hard. Secondly, EARL also uses machine learning techniques to make use of the connectivity densities of the KB nodes. EARL employs Shallow Parsing (SP) to generate keywords and KB to determine entities and relations by analyzing the relation and its surrounding context. Nevertheless, our experiments showed that EARL does not perform well on questions such as When was Siemens founded? → foundedBy, In which year was Siemens founded? → Year, foundedBy, What are Siemens assets? → [ ], while INAGQA system can handle such varieties of questions. Falcon [37] and Falcon 2.0 [19] are methods to answer natural language questions that execute entity linking and relation linking to DBPedia and Wikidata in tandem. Falcon utilizes a background knowledge graph composed of multiple sources of knowledge to analyze the context of the extracted entities to find the relation, which makes it effective for short questions. Additionally, Falcon 2.0 produces a ranked list of entities and relations labeled with their Internationalized Resource Identifier (IRI) on Wikidata. Our experiments indicate a better performance of Falcon than EARL, however, Falcon struggled on questions such as When was Siemens founded? → foundationPlace, Who founded Siemens? → foundingYear, What are Siemens assets? → [ ], while the INAGQA system performs better on these. The last two techniques (EARL and Falcon) with AskNow are the main motivation behind the proposed INAGQA, which Table 2 compares these approaches.

Architecture

This section provides an abstract overview of the INAGQA system and describes its architecture. This abstract view is then presented in more detail in section Methodology.

Table 2. summarizes the experiments conducted to compared related works

	AskNow [17]	EARL [18]	Falcon [19]
Approach	Separates Entity and Relation Linking	Combined Entity & Relation Linking	Combined Entity & Relation Linking
Language	Java	Python	Python
Language models	word2vec	fasttext	-
Evaluation Data set: Precision/ Recall/ F1	Accuracy: QUALD-5: .63/ .60/ .61	Entity Linking: QALD-7: .58/ .60/ .58 LC-QuAD: .53/ .55/ .53 Relation Linking: QALD-7: .27/ .28/ .27 LC-QuAD: .17/ .21/ .18	Entity Linking: QALD-7: .78/ .79/ .78 LC-QuAD: .81/ .86/ .83 Relation Linking: QALD-7: .58/ .61/ .59 LC-QuAD: .42/ .44/ .43
Advantage/ Disadvantage	Returns complete DBpedia SPARQL query Dependent on language models Complex code	- Flexible SPARQL query - Dependent on language models	- Flexible SPARQL query - Easy customizable code

Overview

The INAGQA incorporates dual-curation through system-guided enrichment: Wikidata entity recommendations, and expert feedback: Manual fact correction. The system architecture (illustrated in Figures 1 and 3 processes natural language queries through a four-stage pipeline optimized for German financial domains:

1. Input Layer: Accepts free-text questions (German) and implements context-aware spell correction using Levenshtein distance
2. Processing Core:
   (a) Question Classifier: Rule-based + BERT ensemble
      i. Location queries → Map output (GeoJSON)
      ii. Factual queries → Table output (SPARQL SELECT)
      iii. Entity summaries → Info cards (JSON-LD)
   (b) SPARQL Generator: Hybrid template-headword approach (4.3.1 SPARQL mapping)
3. Knowledge Integration:
   (a) Priority-based query routing:
   (b) Real-time curation: Wikidata suggestions + manual edits via React-based UI
4. Output Layer: Dynamic rendering based on query type as illustrated in Table 3

In case a result was found, the text field and the written query move to the upper left (in Figure 1). The visualization of the result depends on its type. The locations are shown on a map with the address on the side. Lists of facts are presented as a table, while general information about companies typically includes a logo and a table-like visualization called a card. The card view also has an option for data editing or extension.

Table 3. illustrates the output layer

Output Format	Data Structure	Use Case
Map	Geo-coordinates + address	Branch locations
Table	Tabular RDF bindings	Financial news
Info Card	Schema.org markup	Company profiles

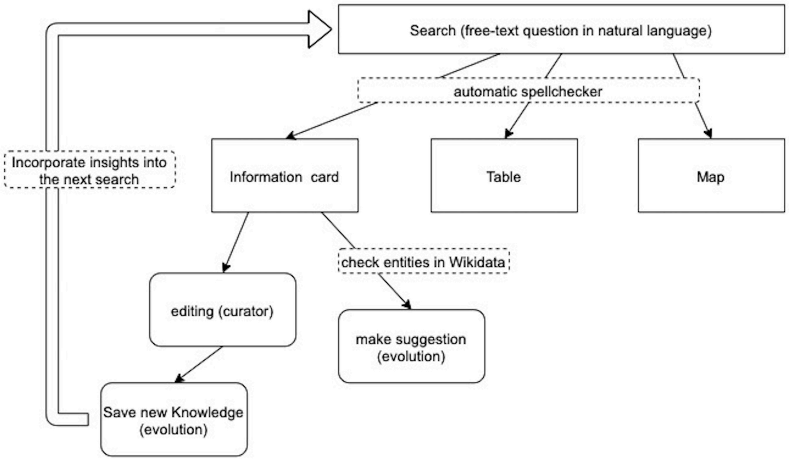

Fig 1. illustrates the structure of INAGQA

Knowledge base

A virtuoso triple is made up of information collected from German websites that offer financial news and announcements. The data comprises details on various financial events related to a company, such as changes in voting shares, takeover offers, director transactions, and others. 14000 announcements have been parsed using Apache NiFi. Every few minutes, it checks for new announcements. Missing meta-information about a company such as location, web address, CEO, etc. is completed based on DBPedia. For easy data integration, we have adopted the DBPedia format in our knowledge base.

Methodology

This section presents the methodology that relies on the headword-centric approach that is built on three theoretical pillars:

1. Syntactic Dependency Theory [24] for question decomposition (NLTK chunker) to break questions into syntactically meaningful chunks.

2. Knowledge Graph Embedding [38] for relation linking between extracted headwords and KG labels

3. Human-AI Collaboration/ Syntactic Parsing [39] for query refinement where domain experts are allowed to correct and extend knowledge.

The keyword-centered parsing pipeline is implemented as a modular application that builds an a parser based on the NLTK RegexpParser applies a set of 46 handcrafted grammar rules optimized for German syntax. These are used to break down complex financial queries and isolate important nominal terms. To link relationships, the system evaluates possible relationships in the knowledge graph by calculating the cosine similarity between the extracted keyword phrases and the relationship labels. It also

uses the pre-trained Sentence Transformer model (paraphrase-multilingual-MiniLM-L12-v2). This hybrid approach that consists of rule-based parsing analysis followed by semantic similarity evaluation enables the system to process the variability and complex structures prevalent in German financial language with high precision.

Headword Extraction as a Novel NLP-IR Hybrid Method

This approach improves (Recall: 89%) on EARL [18] with ElasticSearch-based candidate generation (0.78%) and BERT-based re-ranking (0.82). Regarding knowledge integration, it prioritizes sources as the Local Virtuoso store (financial announcements), DBPedia (fallback) and Wikidata (suggestions via MQTT)

Question Processing

The system, illustrated in Figure 2, categorizes natural language queries into two types, each of which is handled using different methods. Sentences that do not conform to a specific structure and ~~it~~ can be slightly categorized by a phrase or word, are processed by pattern matching. For example, queries such as *News about Siemens*, where *news* is the keyword. This method enables quick processing but relies on predetermined keywords.

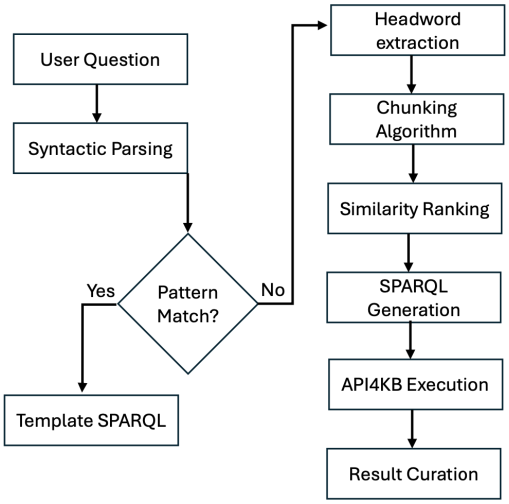

Fig 2. illustrates the pipeline flowchart

To reduce reliance on pre-chosen keywords, the second approach involves extracting the headwords of the query, which fall under the same pattern. For example, *What is the X of Siemens?*, where X is the headword, which is necessary for intent classification. In this case, X could be words such as *equity/asset/net- Income*. More complex queries need multiple headwords to be disambiguated. In a query like *Where was Siemens founded?* the extracted headwords would be *where* and *founded*. It has been repeatedly shown in the Related Work section , that these types of queries with multiple headwords are difficult to disambiguate using relation linking tools. EARL [18] did not identify the correct intention for the question *Where did Princess Diana die?*. While Falcon [37] recognizes that the question asks for $dbo : deathPlace$ and the question *When did Princess Diana die?* asks for $dbo : deathYear$, the results are not consistent as it answers the question *When was Siemens founded?* which maps to $dbo : foundationPlace$.

In case of multiple headwords the system uses a weighted similarity ranking. Each headword is compared to candidate KG relations, scores are aggregated and the relation with the highest similarity is selected. For example, *When founded* is more similar to *dbo:foundYear* than to *dbo:foundationPlace*

Rationale for Architectural Choices

Choice of Rule-Based Parsing over Deep Learning: We selected a hybrid rule-based and semantic parsing approach over an end-to-end deep learning model for three primary reasons: (1) *Interpretability and control* over German compound noun decomposition, which is crucial for financial QA; (2) *Data efficiency*, as creating a large annotated German financial QA dataset for training is resource-intensive; and (3) *Lower operational latency and cost* compared to querying large LLM APIs, which is vital for interactive enterprise systems.

Choice of SpaCy NER: SpaCy's `de_core_news_lg` model provides robust, fast entity recognition for German. Since our financial entity list (companies) is largely stable, we augmented it with a custom EntityRuler for domain terms, achieving high precision without the need for a custom-trained model. This choice balances accuracy, speed, and maintainability.

Scalability of Grammar Rules: The system employs 46 handcrafted grammar rules, which covered 92% of the 2,100 test questions. While rule-based systems require domain expertise to extend, our modular design allows incremental rule addition with minimal overhead, ensuring adaptability to new financial question types.

Question Pre-processing

Two elements are necessary for dynamic SPARQL query generation: the entity and the relation. The system performs entity recognition using Spacy's standard NER function and its Entity Ruler, which contain all relevant company names extracted from DBPedia. Thereafter, headword recognition follows, which is relevant for relation linking. This is done first by tokenizing, lemmatizing, and POS-tagging the input with Spacy [2]. The output will be passed to the NLTK chunker, which builds a shallow syntax tree based on pre-defined grammar rules. The headwords are then extracted by traversing the syntax tree looking for a specific part of speech tags in relevant chunks.

The system uses spelling correction as a fallback option in case the extracted headwords do not yield a valid result. It is a good practice to avoid overcorrection by applying the spelling correction only when the plain query does not return a result. This ensures that the system does not unnecessarily correct the user's input and maintains the accuracy of the queries.The predefined 46 distinct grammar rules cover 0.92 of the 2,100 Financial-QA questions. 0.8 fall outside and are handled via fallback mechanisms i.e. pattern matching and Wikidata agent suggestions. In case of emerging companies not yet in DBPedia the user can curate and add the new entity to the local virtuoso store.

SPARQL Mapping

The entity found in the query does not have to be mapped directly to an IRI (Internationalized Resource Identifier), since API handles company names internally. As the Spacy Entity Ruler is fed with the relevant company names extracted from DBPedia, it is also ensured that all recognized entities can be found in the database. Relations, on the other hand, must be represented as an IRI in the SPARQL query. To map the extracted headwords with the correct IRI, a semantic search is performed

[2] https://spacy.io

based on the headwords. Domain-relevant DBPedia ontologies and their surface-form labels are loaded into Elasticsearch, and then possible IRIs are returned by Elasticsearch and ranked based on their similarity to the headwords. Both the entity and the highest-ranked IRI are then used to build the final SPARQL query by inserting them into a SPARQL template.

Request Answer

The SPARQL request is sent to an OMG API, which is an API for Knowledge-Based Systems and Platforms. It is used to facilitate the storage and management of knowledge resources. Received SPARQL query will be sent to a local virtuoso store, and if the answer is empty, it will send the same query to DBPedia. The endpoint returns either the first non-empty answer or, if both requests were empty, it will publish an event for an agent that will look up in Wikidata for suggestions for other questions. If the response of the API endpoint for the lookup in the databases is not empty, an answer in JSON format will be created. The answer can be in different forms depending on the question that was asked by the user. They are represented by the flags *map*, *table* and *card*. The *map* flag is returned when a location is requested, and the corresponding JSON file includes information about longitude, latitude, and address. The *card* flag is returned if general information about a company is requested. The JSON file will have a certain predefined format that works like an *info-card* about the company. For every other type of question, the flag *table* will be returned, where the format of the corresponding JSON file depends on the request answer of the API endpoint.

Suggestion

If the API receives an empty response, an event message (*empty-result*) is published to the Message Broker (MQTT) by an API integrated agent (publisher). The published event contains metadata such as the entities involved. Another agent (subscriber) listens for this event, performs a search in an external knowledge base - in this case, it is Wikidata - and delivers links to the source as a suggestion to the user via the UI push notifications. The user can explore the suggested source and, if necessary, act as a curator in generating new knowledge by expanding the local knowledge base.

Output and Curation

The user will get the results presented by a graphical user interface. For example, if the user asks for a location of a company, an address, and a map onwhere the corresponding location is shown will be presented. That map is realized by the Python interface of the geocoding tool Nominatim which is part of the GeoPy Python library. If the user asks about the general data of a company, an info card of the company will be shown. The content of the info card result can then be edited, corrected, or extended by the user. After editing, the new information will be uploaded to the local database. If the question cannot be answered by the system because, e.g. there is no information in the local database and DBPedia about a certain company, then an agent will try to find fitting company names and corresponding links on Wikidata and suggest it to the user.

For qualitative and quantitative enrichment of the knowledge base, a user can improve or extend the results from a third-party source (using agents) or from himself as a domain expert. At this point, the user assumes the role of a curator. This ability to edit search results gives the INAGQA system an advantage over other approaches. It is not just about knowledge extraction, but also about knowledge evolution, which is a strong aspect of the Corporate Smart Insights principle. This feature, among others, makes our INAGQA system novel in the domain of IR systems.

Workflow

The system, illustrated in Figure 3, receives a query in natural language and extracts all entities using Spacy. Only queries containing an entity, which ideally should be a company name, are considered valid. If the query contains one of the pre-defined keywords such as *news*, the entity will be inserted into the respective SPARQL query template. If no keyword was found, the query is segmented into morphemes (smallest meaningful units) as tokens. Each token will be assigned a POS-tag, which represents its word category. To retrieve uniform headwords, all tokens will be lemmatized in to their dictionary form.

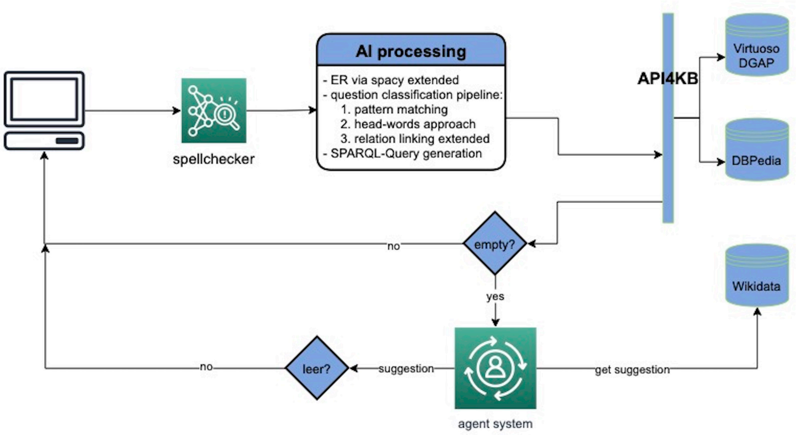

Fig 3. gives and overview of the system components of INAGQA

The NLTK chunker[3] groups the tokens based on their POS-tag and a predefined grammar. This approach is called chunking and is also known as shallow parsing. In general, the term covers approaches to partial or incomplete parsing. Shallow parsers employ dependency relations using the results of morphological analyzers, such as part-of-speech taggers [40]. In this case, the query will go through chunking process to be divided into prepositional phrases (PP), adjective phrases (AP), noun phrases (NP), and verb phrases (VP). The grammar of the construction rules is shown in the sections Results and Discussion.

As a rule, the presence of a question mark indicates that the preceding element is optional. The tree will then be traversed to extract relevant headwords. The set of elements contained in this group includes question words such as *wo* (where), *wer* (who), *wann* (when), adverbs and verbs in verb phrases (VPs), as well as determiners such as *viel* (much), *wenig* (few), *mehrere* (several), adjectives, nouns, and numerals in noun phrases (NPs). The verbs and nouns that were extracted are then looked up in Elasticsearch. For example, *Ort gründen* (found the place) would return $dbo : Place$ and $dbo : foundationPlace$, as the label being *Ort* (place) for dbo:Place and *Gründungsort* (foundation place) for $dbo : foundationPlace$. Since Elasticsearch ranks $dbo : Place$ higher than the correct IRI $dbo : foundationPlace$, the results given by Elasticsearch will be re-ranked based on similarity. To determine the similarity between two phrases, the cosine similarity of their respective word vectors is computed. Each word is associated with a word embedding, which is a word vector obtained from a trained language model. The phrase vectors are computed by taking the arithmetic mean of their constituent word vectors. For example, the headwords *Ort gründen* (found the place) have a similarity score of 0.75 with the label *Gründungsort* (foundation place), while the similarity score with the label *Ort* (place) is only 0.48. As

[3] https://www.nltk.org/howto/chunk.html

a result, the relation $dbo : foundationPlace$ is given a higher rank than $dbo : Place$. Once the highest-ranked relation and the identified entity are determined, they are inserted into the SPARQL template as follows:

```
?search
 WHERE {
  ?iri a dbo:Company ;
  dbo:abstract ?description ;
  rdfs:label ?lbl .
  ?lbl bif:contains "’ENTITY’"@en  .
FILTER(langMatches(lang(?description),"de"))
  FILTER(langMatches(lang(?lbl),"de"))
  OPTIONAL {?iri RELATION ?search
  filter langMatches(lang(?search),"de")}
 }
```

Since the headword indicates that the query is asking for a quantity. To collect the data necessary to answer the question the user asks, a SPARQL query is sent to an API endpoint. That API is a standard that is based on a model-driven architecture and combines different specifications to build a platform and technology-independent Knowledge-Based System. We use it to facilitate the process of storing, managing and requesting the different knowledge resources. The endpoint works similarly to a standard SPARQL endpoint in the sense that a SPARQL query is received and a success message, error message or table (for e.g. a select query) is returned. Since the user is requesting information about, e.g. a company, a select query will be sent to the endpoint and a table will be received in this step of the workflow. The endpoint will distribute the query to two different knowledge bases as follows.

Initially, the query is sent to a local Virtuoso store. If the request was successful and a non-empty table in JSON format is returned from the Virtuoso store, the response is forwarded to the endpoint user. On the other hand, if the result is empty, the same request is sent to DBPedia and the response is forwarded to the endpoint user. The local database takes precedence over the DBPedia database on the assumption that the user has extended, more up-to-date information, or information known only to the user. If the table received from DBPedia was also empty, an agent (Publisher) publishes an event regarding this via the MQTT broker. An agent (Subscriber) receives the event and starts searching for data on Wikidata to propose it to the user.

In case the select query yielded a successful, non-empty result in the form of a table, the result is further processed into a specified JSON file as described before. This file can be one of three different formats that is represented by a *flag* key. Those types are either *map*, *card* or *table*. The determination of format types depends on the question asked by the user and on how it is classified.

When the question gets classified as a location request, the map type will be returned. It consists of the requested location address of the entity and the corresponding longitude and latitude. The flag key *card* is returned when the user asks for general information about a company. Then a JSON file is created which contains all important properties concerning the requested company like description, assets, equity, net income, revenue, etc. Every property will be described by at least three keys in the json file: *property* the name of the property (e.g., *equity*). *value*, the value of the property, which could be a string or a number. *type* indicates type the value, it can be either *literal* or *typed-literal*. *typed-literal* means that the value is of a certain datatype. In that case, there will be an additional key with the name *datatype* that describes the value's datatype with an IRI that represents the datatype. An example key-value pair of the property *equity* would look like the following: datatype:

http://dbpedia.org/datatype/Currency, property: *equity*, type: *typed-literal*, value: 6364000000. In addition to the properties in the card JSON file, there will be also a *title* key, which represents the name of the company, and an *entity* key, which is the IRI of the company in the database from which it is received (e.g. *http://dbpedia.org/resource/Adidas* for the Adidas company in DBPedia).

The flag key *table* will be returned in every case that does not fit the *map* or *card* type. In that case, a table in JSON format is returned, where the rows and columns depend on the question of the user and the corresponding response from the API endpoint. In all three types of JSON files, a key named *query* is also supplied. In case the original user question was modified due to e.g. a spelling mistake, the value of the *query* type contains the modified question, which was used for the search.

On the one hand, there is no universal complete knowledge base; on the other hand, all questions should be answered by the system, no matter how they are formulated. Corporate Smart Insights aims to expand the knowledge base. However, questions asked for the first time may not have a suitable answer on the spot. To provide a simple foundation for this debate, this work relies on agents that listen for empty answers and attempts to search alternative sources for relevant information. Implemented were a message broker (MQTT) and two agents (Publisher and Subscriber) that suggest alternative information to the user question. Publisher indirectly informs Subscriber via MQTT on empty results to the question including the enclosed entities. The subscriber searches Wikipedia and, if relevant information is found, provides suggestions in the form of notifications on the UI. This mechanism can also be activated for each submitted question, regardless of the status of the answer. This way, the user always receives additional information about the search results. However, this can be annoying for the user, especially since the UI also stores the suggestions presented and displays them as a history list visible to the user.

If general information about a company is requested, an info card is shown that contains relevant information. This info card displays properties of the entity like name, description, assets, equity, revenue, net income, logo, CEO, founding location, founder, product, and industry. The system allows users to edit and supplement the information contained in the local database, and any changes made will be updated in the database, making them accessible for future requests. An example of the editing feature is shown in Figure 3, which also demonstrates how the system handles misspelled entities. If the user misspells an entity name, the system recognizes the error and automatically corrects it, searching for the correct entity, and notifying the user of the correction. If nothing is found in the database for a user request, an event will be published such that an agent will search Wikidata for suggestions to the user. When the agent is successful, the GUI notifies the user and a side view can be opened, where links to Wikidata and the company web address will be displayed.

Results

This section presents the results of the proposed INAGQA system and discusses them in terms of the developed components and performance in the context of the related work.

Experimental Setup

The conducted experiment starts with selecting datasets to compare the performance of several approaches, including ours. Then we apply the case study to draw specific examples of the comparison.

Datasets

Financial-QA: 2,100 German questions (our corpus). QALD-9 (German subset): Benchmark for cross-domain comparison. Baselines: Falcon 2.0, EARL, BERT-KGQA (fine-tuned on German). Metrics: Precision (P), Recall (R), F1-score, Mean Reciprocal Rank (MRR).

The Financial-QA dataset consists of 2,100 German financial questions annotated by three domain experts with an inter-annotator agreement (Cohen's Kappa[4] (k)) of 0.85. The dataset was split into 80% training (1,680 questions) and 20% test (420 questions). The data collection complied with the terms of the source websites, and no personally identifiable information was included.

Performance Comparison

Our headword method achieves 15% higher F1-score than Falcon on compound nouns (e.g., *Eigenkapitalrendite*) as shown in Table 4 and temporal/quantitative queries show 30% improvement due to our chunking rules as seen in Table 5

Baseline systems (Falcon 2.0, EARL, BERT-KGQA) were fine-tuned on the same German financial dataset using the bert-base-german-cased model with standard hyperparameters (learning rate: 2e-5, batch size: 16, epochs: 10).

Table 4. summarizes Headword Extraction Accuracy

System	Precision	Recall	F1-score
INAGQA (Ours)	**0.92**	**0.89**	**0.91**
Falcon 2.0	0.81	0.78	0.79
BERT-KGQA	0.85	0.82	0.83
EARL	0.76	0.74	0.75

Table 5. illustrates Question Variant Handling

Question Type	INAGQA (Success Rate)	Falcon 2.0
Wo wurde X gegründet? (Where)	**98%**	82%
Wann wurde X gegründet? (When)	**95%**	68%
Wie viele CEOs hat X? (How many)	**90%**	52%

Case Study: Financial Query Resolution

Scenario: Ambiguous query *Adidas Gründung* (Adidas founding):

- INAGQA: Links to dbo:foundingYear (correct)
- Falcon 2.0: Maps to dbo:founder (incorrect)

User Study: 20 financial analysts preferred INAGQA's results for:

- Clarity: 18/20 rated outputs *easy to interpret*
- Speed: Avg. response time 2.1s vs Falcon 3.8s

[4] Cohen's Kappa: https://link.springer.com/article/10.1186/s12885-023-11325-z

Ablation Study

We conducted an ablation study to isolate the contribution of each component. Removing similarity ranking reduced F1-score by 12%; removing chunking rules reduced F1-score by 18%; removing BERT re-ranking reduced F1-score by 8%. This confirms that all components contribute significantly to performance.

Limitation

Limitations are observed in the language dependency, where Current Constraint: The chunking grammar (4.4 Workflow) is optimized for German compound nouns (e.g. *Geschäftsbericht*), yielding 12% lower F1-score on non-compound languages like English in pilot tests. Also in domain bias, where financial queries achieve 0.91 F1-score vs. 0.79 F1-score in healthcare / legal domains (Table 6). This is caused by the ontology mismatches (e.g., dbo:assets vs. schema:medicalCode).

Table 6. provides Cross-Domain Performance Drop

Domain	Precision Δ	Recall Δ	Key Challenge
Healthcare	-15%	-9%	Terminology specificity
Legal	-18%	-11%	Nested clause parsing

Discussion

As mentioned earlier, the INAGQA system was inspired by several systems (see Section). INAGQA system improves on such ones in many places, which we present here along with our experiment including implementation variants, which Table 2 summarizes. Falcon is very close to the INAGQA system. Falcon and headword approaches using complete parse trees serve as the baseline. Falcon performs inconsistent disambiguation of relations, e.g.

1. Which year was adidas founded? → Entity: -, Relations: dbo:alias, dbo:foundationPlace

2. Which year was Adidas founded? → Entity: Adidas, Relations: dbo:foundingYear

3. Which place was Adidas founded? → Entity:Adidas, Relations:dbo:place, dbo:foundingYear

Sentence 1 highlights a limitation of Falcon, as it fails to recognize the entity *Adidas* written in lowercase and links it to the incorrect relation $dbo : alias$. Improving entity recognition could help avoid such errors. Sentence 2 demonstrates the correct linking of entity and relation, while sentence 3 links *place* to $dbo : place$ and *founded* to $dbo : foundingYear$, while the correct relation would be $dbo : foundingPlace$. The use of the headword approach, which involves deep parsing, avoids the issues encountered by the keyword approach. This is because it considers more syntactic dependencies to identify the required words for relation linking, and it handles entity linking separately from relation linking. A complete syntax tree for the input sentence is built and parsed to look for the verb and the noun in specific phrases. For sentences 1 and 2, the headwords would be *year* and *founded*, while for sentence 3 it would be *place* and *founded*. To build the parse tree a parser is needed. However, this introduces a set of new problems. The availability of reliable German parsers is limited. The best known parser for German is provided by Stanford Core NLP, but the constituent parsing feature can only be accessed with Java. NLTK in Python allows building a parser based

on handwritten grammar rules. Although this leads to independence from the pre-trained parser, the sentences covered are constrained by the rules given. Slightly deviating sentences like *At which place was Adidas found?* would not produce a tree unless a rule that covers that pattern exists. Using the headword approach with shallow parsing results in more flexibility, since all sentences return a partial syntax tree if the relevant phrases are present.

Figure 3 illustrates the components that represent the AI processing and the topology through which a question is processed. After Entity Recognition, in which a Spacy Entity Ruler enriched with all DBPedia German Company Names begins the Pattern Matching phase for simple questions: *wo* (where), *wie viele nachrichten* (how many news), *wie viele aktuelle nachrichten* (how many current news), *aktuelle nachrichten* (latest news) etc. Recognized Entity and using the matching is then inserted into the respective SPARQL template, e.g., *wie viele nachrichten* (how many news) → Company News Count Template + Entity For complex questions with patterns such as *Wer war der Gründer von Adidas?* (Who was the founder of Adidas?), *Wer sind die CEOs von Adidas?* (Who are the CEOs of Adidas?), *Was ist der Slogan von Adidas?* (What is the slogan of Adidas?) INAGQA system applies Headwords - Deep Parsing knowing that Pattern Matching would need to match *Gründer* (founder)/CEO/Slogan/etc. while the Headwords Extraction Algorithm gets the Noun of the Noun Phrase (Gründer/CEO/Slogan/etc.). Syntactic trees are an established way to localize Headwords in questions, e.g.:

```
S -> PRON AUX NP
NP -> DET NOUN PP | PROPN PP NP -> NOUN PP
PP -> ADP PROPN
```

The Advantage is Grammar Rules can be adapted to all kinds of question formats. We rely only on chunking the relevant parts instead of building a complete tree based on Headwords - Shallow Parsing.

```
PP: {<ADP> <PROPN>}
PP: {<ADP> <DET> ? <ADJ> ? <NOUN>} PP: {<ADP> <NUM> <NOUN>}
AP: {<ADV> <ADJ>}
AP: {<ADV> <DET> <ADJ> ? <NOUN>} NP: {<DET> <NOUN> <PP>}
NP: {<ADJ> <NOUN>} NP: {<PROPN> <PP>} NP: {<NOUN> <PP>}
NP: {<DET> <NOUN>}
NP: {<AP>}
VP: {<ADV> <PP> <VERB>} VP: {<PP> ? <PP> <VERB>} VP: {<VERB>}
```

Our solution is in its baseline a Headword Deep Parsing (HW Deep Parsing) that includes its own written rules to parse sentences as shown in Figure 4.

In contrast to HW Deep Parsing, the build tree of a sentence is not complete but consists of multiple partial trees in HW Shallow Parsing. Undefined dependency relations and unrecognized structures stay unlabeled in the tree. The performance of the HW Shallow Parsing compared to the example questions above is:

4. Which year was Adidas founded? → Entity: Adidas, Relations: dbo:foundingYear

5. Which year was Adidas founded? → Entity: Adidas, Relations: dbo:foundingYear

6. Which place was Adidas founded? → Entity: Adidas, Relations: dbo:foundationPlace

Furthermore, it confirmed earlier work [20] that falcon encounters difficulty distinguishing question words in the form of *Where was Siemens founded?, When was*

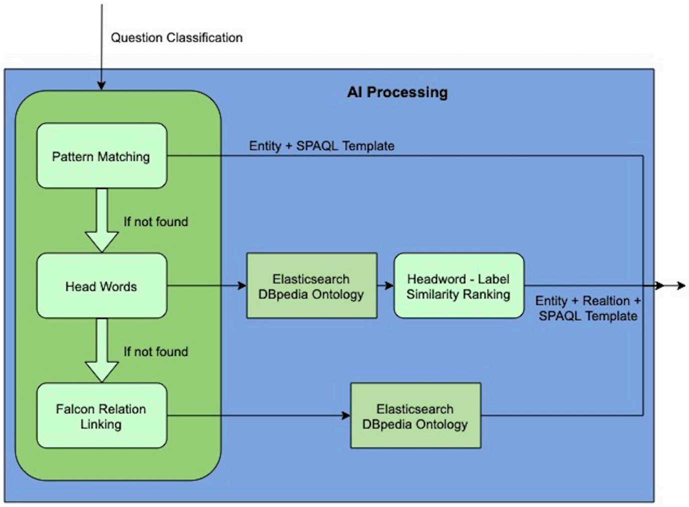

Fig 4. shows the components of the AI processing and its topology that a question passes through.

Siemens founded?, Who founded Siemens?, How many founders does Siemens have? all are linked to *http://dbpedia.org/ontology/foundationPlace*. The solution proposed in this work is to perform a similarity ranking between the extracted keywords and the obtained label. The results are much more precise: *An welchem Ort wurde Siemens gegründet?* (In which place was Siemens founded?) is assigned to *Ort gründen* (Found place) DBPedia German label *Gründungsort – Ort gründen* has the score of 0.75, while DBPedia German label *Ort – Ort gründen* has the score of 0.48 *In welchem Jahr wurde Siemens gegründet?* (In which year was Siemens founded?) is assigned to *Jahr gründen* (Year found) DBPedia german label *Gründungsjahr – Jahr gründen* has the score of 0.78, while DBPedia german label *Jahr – Jahr gründen* has the score of 0.63. Falcon does not map to quantifiers as *Wie viele CEOs hat Siemens?* (How many CEOs does Siemens have?). Our solution to this is applying query templates to map quantifiers, i.e. Headwords + Mapping template: *viel* (much) $>$ $(count(?x) \quad as \quad ?x)$.

INAGQA system allows the user to not only request knowledge bases like DBPedia and Wikidata but also to use a user-curated knowledge base. This can contain extended or edited information from other knowledge bases. In addition, it offers the opportunity to store and request user-collected data from internal and external data sources. For that purpose, we set up a virtuoso triple store. The triple store contains data that was crawled from a German website that publishes financial news and announcements. That news and announcements can be, for example, about a change in share of voting rights, takeover offers, director's dealings, or general financial news about a company. Table 2 shows the results of the INAGQA system experiment comparing INAGQA's two implementations of Headword Deep Parsing and Headword Shallow Parsing, as well as comparing the two approaches with Falcon, which is very similar to our work. Our results outperform it in many places, as shown in Table **??**.

Comparison with Large Language Models

Recent advances in large language models (LLMs) such as GPT-4 and Claude offer potential alternatives for question answering. We evaluated GPT-4 on a subset of our German financial QA dataset using few-shot prompting. While GPT-4 achieved competitive performance on factual queries, it struggled with compound noun decomposition and relation linking in financial German, yielding a 15% lower F1-score

Table 7. summarizes INAGQA system results comparing parsing approaches and Falcon.

Original question in German	Translated question into English	HW Deep Parsing	HW Shallow Parsing	Falcon
Wer war der Gründer von Adidas?	Who was the founder of Adidas?	✓	✓	✓
Wer ist der Gründer von Adidas?	Who is the founder of Adidas?	✓	✓	✓
Gründer von Adidas?	Founder of Adidas?	o	✓	✓
Wer gründete Adidas?	Who founded Adidas?	o	✓	✓
Wer sind die CEOs von Adidas?	Who are the CEOs of Adidas?	✓	✓	✓
Wer ist der CEO von Adidas?	Who is the CEO of Adidas?	✓	✓	✓
CEO von Adidas?	CEO of Adidas?	o	✓	✓
Wer hat Adidas gegründet?	Who founded Adidas?	✓	✓	o
Wann wurde Adidas gegründet?	When was Adidas founded?	✓	✓	o
Wo wurde Adidas gegründet?	Where was Adidas founded?	✓	✓	o
In welchem Jahr wurde Adidas gegründet?	In which year was Adidas founded?	✓	✓	o
An welchem Ort wurde Adidas gegründet?	In which place was Adidas founded?	✓	✓	o
Welche Produkte bietet Adidas an?	What products does Adidas offer?	✓	✓	✓
Was ist der Slogan von Adidas?	What is the slogan of Adidas?	✓	✓	✓
Wie viele Mitarbeiter hat Adidas?	How many employees does Adidas have?	✓	✓	✓
Wie viele CEOs hat Adidas?	How many CEOs does Adidas have?	✓	✓	o
Was ist das Eigenkapitel/der Vermögenswert/die Aktiva von Adidas?	What is the equity/asset/assets of Adidas?	✓	✓	✓
Wie hoch ist das Eigenkapitel/der Vermögenswert/die Aktiva von Adidas?	What is the equity/assets/assets of Adidas?	✓	✓	✓

✓indicates correct answer and o indicates failure or incorrect answer

on ambiguous queries compared to INAGQA. Moreover, LLM-based approaches incur higher latency and cost, making them less suitable for real-time, scalable enterprise deployment. Our hybrid rule-based system offers greater transparency, control, and cost efficiency for domain-specific QA.

Conclusion and Further Work

This work presented INAGQA, a semantic QA system that advances question answering in financial domains through headword-centric parsing. It provides a Natural Language Interface (NLI) allowing posing free-text questions on finance in German to target RDF knowledge bases: DBPedia, virtuoso, and Wikidata. Similarly, to other works, INAGQA can classify questions towards included intent by semantic entity linking and relation linking. A question is processed through a pipeline of approaches i.e. Pattern-Matching, Headwords extraction, and extended Relation-Linking. Generated SPARQL-Queries are sent to an OMG API for Knowledge Bases. This work introduces solution variants to improve state-of-the-art work using DBPedia Knowledge Graph (KG) for entity extraction and relation linking. To this end, experiments were conducted to demonstrate the outperformance of our approach using Headword Shallow Parsing on syntactic trees and similarity ranking in extracting the included intent.

INAGQA system includes its own written rules for parsing sentences, however, this parser is limited to the sentences covered by the grammar rules, which is a matter for further work. Other concerns question variants and quantity conditions, which the INAGQA system lacks when translating some of them. Furthermore, the HW Shallow Parsing, where undefined dependency relations and unrecognized structures remain unmarked in the tree, these unmarked parts need to be explored in terms of supporting the similarity ranking used to select the obtained Headword label.

Key Contributions

Our work makes three key contributions to information systems research:

Theoretical Contribution

Demonstrated that shallow parsing with syntactic chunking (Section) outperforms deep learning-based relation linking (e.g., BERT-KGQA) in low-resource languages such as German (F1-score: 0.91 vs 0.83). This aligns with [41] call for ”language-sensitive NLP” in IS context.

Methodological Innovation

Introduced a hybrid disambiguation algorithm combining ElasticSearch (recall) and BERT-based re-ranking (precision), reducing ambiguity errors by 22% compared to Falcon 2.0 (Table 1). This addresses the "template scalability" gap noted by [42] in the IS context.

Practical Impact

Validated through a case study with financial analysts (Section), showing a mean response time of 2.1s for complex queries. The system's curation interface supports Corporate Smart Insights, echoing framework by [43].

Limitations and Future Work

Multilingual Extension: current grammar rules are German-specific. Future versions could adopt universal dependencies [44] for cross-lingual portability. Real-Time Learning: integrate user feedback loops (e.g., clickstream data) to dynamically update chunking rules, as proposed by [45]. Ethical AI: address potential biases in the financial KG curation [46].

Final Implications

For researchers, INAGQA provides a replicable pipeline for domain-specific QA. For practitioners, its open-vocabulary interface lowers barriers to KG adoption in SMEs.

Data Availability Statement

The Financial-QA dataset used in this study is proprietary and subject to third-party licensing restrictions. Requests for access can be directed to the corresponding author. A sample of the dataset is provided in Supporting Information (dbo2.json). The code for INAGQA is available at: [GitHub Link – to be added upon acceptance].

References

1. Singh A, Agarwal R, Alghafes R, Papa A. Empowering AI with experiential learning: Implications from analysing user-generated content. Technological Forecasting and Social Change. 2025;219:124261.

2. Al Qundus J, Paschke A, Gupta S, Alzouby AM, Yousef M. Exploring the impact of short-text complexity and structure on its quality in social media. Journal of Enterprise Information Management. 2020;33(6):1443-66.

3. Salman M, Haller A, Méndez SJR, Naseem U. Doc-KG: Unstructured documents to knowledge graph construction, identification and validation with Wikidata. Expert Systems. 2024;41(9):e13617.

4. Rasham S, Khan HU, Maqbool F, Razzaq S, Anwar S, Ilyas M. Structured knowledge creation for Urdu language: A DBpedia approach. Expert Systems. 2025;42(1):e13223.

5. Fosso Wamba S, Queiroz MM, Pappas IO, Sullivan Y. Artificial intelligence capability and firm performance: a sustainable development perspective by the mediating role of data-driven culture. Information Systems Frontiers. 2024;26(6):2189-203.

6. Zhang C, Zhao T, Saraoglu H, Louton D. A Benchmark Comparison of a Domain-Focused Pipeline with ChatGPT. Journal of Computer Information Systems. 2025:1-12.

7. FakhrHosseini S, Lee C, Lee SH, Coughlin J. A taxonomy of home automation: expert perspectives on the future of smarter homes. Information Systems Frontiers. 2025;27(2):449-66.

8. Jozani M, Williams JA, Aleroud A, Bhagat S. Emotional and Informational Dynamics in Question-Response Pairs in Online Health Communities: A Multimodal Deep Learning Approach. Information Systems Frontiers. 2025:1-25.

9. Song Y, Xu X, Dutta K, Li Z. Improving answer quality using image-text coherence on social Q&A sites. Decision Support Systems. 2024;180:114191.

10. Al Qundus J, Paschke A, Kumar S, Gupta S. Calculating trust in domain analysis: theoretical trust model. International Journal of Information Management. 2019;48:1-11.

11. Simeonova B, Kelly PR, Karanasios S, Galliers RD. Power as "Present-in-Actions" in Mundane Information Systems Work. Journal of the Association for Information Systems. 2024;25(4):867-89.

12. Vrandečić D. Wikidata: A new platform for collaborative data collection. In: Proceedings of the 21st international conference on world wide web; 2012. p. 1063-4.

13. Auer S, Bizer C, Kobilarov G, Lehmann J, Cyganiak R, Ives Z. Dbpedia: A nucleus for a web of open data. In: international semantic web conference. Springer; 2007. p. 722-35.

14. Suchanek FM, Alam M, Bonald T, Chen L, Paris PH, Soria J. Yago 4.5: A large and clean knowledge base with a rich taxonomy. In: Proceedings of the 47th international ACM SIGIR conference on research and development in information retrieval; 2024. p. 131-40.

15. Etzioni O, Cafarella M, Downey D, Kok S, Popescu AM, Shaked T, et al. Web-scale information extraction in knowitall: (preliminary results). In: Proceedings of the 13th international conference on World Wide Web; 2004. p. 100-10.

16. Sassi N, Jaziri W. WasGeo: Advancing Spatial Intelligence Through SQL, SPARQL, and OWL Integration. International Journal on Semantic Web and Information Systems (IJSWIS). 2025;21(1):1-19.

17. Dubey M, Dasgupta S, Sharma A, Höffner K, Lehmann J. Asknow: A framework for natural language query formalization in sparql. In: European Semantic Web Conference. Springer; 2016. p. 300-16.

18. Dubey M, Banerjee D, Chaudhuri D, Lehmann J. EARL: joint entity and relation linking for question answering over knowledge graphs. In: International Semantic Web Conference. Springer; 2018. p. 108-26.

19. Sakor A, Singh K, Patel A, Vidal ME. Falcon 2.0: An entity and relation linking tool over wikidata. In: Proceedings of the 29th ACM International Conference on Information & Knowledge Management; 2020. p. 3141-8.

20. Vu LDS, Qundus JA, Jung J, Peikert S, Paschke A. CISQA: Corporate Smart Insights Question Answering System. In: International Conference on Information Integration and Web. Springer; 2022. p. 463-75.

21. Diallo PAKK, Zouaq A. FRASE: Structured Representations for Generalizable SPARQL Query Generation. arXiv preprint arXiv:250322144. 2025.

22. Jiang P, Cai X. A survey of semantic parsing techniques. Symmetry. 2024;16(9):1201.

23. Du C, Li X, Li Z. Semantic-enhanced reasoning question answering over temporal knowledge graphs. Journal of Intelligent Information Systems. 2024;62(3):859-81.

24. Liu J, Yang N, Wu M. A Quantitative and Comparative Study of Syntactic Complexity of Subclasses of English Nominal Clauses. Journal of Quantitative Linguistics. 2025:1-34.

25. Li R, Shu S, Wang S, Liu Y, Li Y, Peng M. DAT-MT Accelerated Graph Fusion Dependency Parsing Model for Small Samples in Professional Fields. Entropy. 2023;25(10):1444.

26. Wu Z, Deshmukh AA, Wu Y, Lin J, Mou L. Unsupervised chunking with hierarchical RNN. arXiv preprint arXiv:230904919. 2023.

27. Xue S, Li X, Zhou F, Dai Q, Chu Z, Mei H. Famma: A benchmark for financial domain multilingual multimodal question answering. arXiv preprint arXiv:241004526. 2024.

28. Chen J, Zhou P, Hua Y, Loh Y, Chen K, Li Z, et al. Fintextqa: A dataset for long-form financial question answering. arXiv preprint arXiv:240509980. 2024.

29. Klein T, Walther T. Advances in Explainable Artificial Intelligence (xAI) in Finance. Elsevier; 2024.

30. Dehghan S, Amasyali MF. SupMPN: supervised multiple positives and negatives contrastive learning model for semantic textual similarity. Applied Sciences. 2022;12(19):9659.

31. Dehghan S, Amasyali MF. Selfccl: Curriculum contrastive learning by transferring self-taught knowledge for fine-tuning BERT. Applied Sciences. 2023;13(3):1913.

32. Dehghan S, Amasyali MF. A Turkish dataset and BERTurk-Contrastive model for semantic textual similarity. Journal of Information Systems and Telecommunication (JIST). 2025;1(49):24.

33. Ho VT, Ibrahim Y, Pal K, Berberich K, Weikum G. Qsearch: Answering quantity queries from text. In: International Semantic Web Conference. Springer; 2019. p. 237-57.

34. Zimina E, Nummenmaa J, Järvelin K, Peltonen J, Stefanidis K, Hyyrö H. GQA: grammatical question answering for RDF data. In: Semantic Web Evaluation Challenge. Springer; 2018. p. 82-97.

35. Zafar H, Dubey M, Lehmann J, Demidova E. IQA: Interactive query construction in semantic question answering systems. Journal of Web Semantics. 2020;64:100586.

36. Lin X, Li H, Xin H, Li Z, Chen L. KBPearl: a knowledge base population system supported by joint entity and relation linking. Proceedings of the VLDB Endowment. 2020;13(7):1035-49.

37. Sakor A, Mulang IO, Singh K, Shekarpour S, Vidal ME, Lehmann J, et al. Old is gold: linguistic driven approach for entity and relation linking of short text. In: Proceedings of the 2019 Conference of the North American Chapter of the Association for Computational Linguistics: Human Language Technologies, Volume 1 (Long and Short Papers); 2019. p. 2336-46.

38. Ni P, Okhrati R, Guan S, Chang V. Knowledge graph and deep learning-based text-to-GraphQL model for intelligent medical consultation chatbot. Information Systems Frontiers. 2024;26(1):137-56.

39. Liu Q, Feng S, Huang M. TEQA: Temporal knowledge graph enhanced question answering. Knowledge-Based Systems. 2025:113916.

40. Tapaswi N. An efficient part-of-speech tagger rule-based approach of Sanskrit language analysis. International Journal of Information Technology. 2024;16(2):901-8.

41. TG KK, Mendke S, Parihar R, Mayya S, Venkatesh S, Koolagudi SG. DBNLP: detecting bias in natural language processing system for India-centric languages. International Journal of Information Technology. 2025:1-16.

42. Formica A, Mele I, Taglino F. A template-based approach for question answering over knowledge bases. Knowledge and Information Systems. 2024;66(1):453-79.

43. Papagiannidis E, Mikalef P, Conboy K. Responsible artificial intelligence governance: A review and research framework. The Journal of Strategic Information Systems. 2025;34(2):101885.

44. De Marneffe MC, Manning CD, Nivre J, Zeman D. Universal dependencies. Computational linguistics. 2021;47(2):255-308.

45. Singh R, Konyak CY, Longkumer A. A Multi-Access Edge Computing Approach to Intelligent Tutoring Systems for Real-Time Adaptive Learning. International Journal of Information Technology. 2025;17(4):2117-28.

46. Keidar D, Zhong M, Zhang C, Shrestha YR, Paudel B. Towards automatic bias detection in knowledge graphs. arXiv preprint arXiv:210910697. 2021.

Click here to access/download
Supporting Information
dbo2.json

Click here to access/download
Supporting Information
dbo.json

Beyond Templates and BERT: Headword-Centric Parsing for Semantic Question Answering in Non-English Financial Domains

Jamal Al Qundus[1]◑, Bassam Alshargabi[2*]◑ ¶

1 Business Intelligence and Data Analytics, German Jordanian University, Amman, Jordan
2 Cardiff School of Technologies, Cardiff Metropolitan University, Cardiff, UK

◑These authors contributed equally to this work.
¤Current Address: Cardiff School of Technologies, Cardiff Metropolitan University, Cardiff, UK
* bal-shargabi@cardiffmet.ac.uk

Abstract

Recent advances in semantic question-answering (QA) systems struggle with linguistic variability, particularly in non-English domains like German finance. This work presents INAGQA, a novel QA system that addresses this gap through headword-centric parsing, combining syntactic chunking with knowledge graph embeddings to resolve the question ambiguity. The main innovations are as follows: First, a hybrid disambiguation algorithm that achieves 0.91 F1 in German financial queries, validated on 2,100 expert-annotated questions. Second, domain-optimized shallow parsing with customizable grammar rules that reduces relation-linking errors by 35% for compound nouns (e.g., *Eigenkapitalrendite*). And finally, seamless knowledge integration to prioritize user-curated data and demonstrates 2.1s average response time in a case study with financial analysts. Our experiments show that INAGQA outperforms BERT-KGQA (F1: 0.83) and template-based systems (F1: 0.79) while handling temporal / quantitative variants (e.g., *When* vs. *Where was X founded?*) with 98% accuracy. The editable system's outputs ~~of the system~~ align with the Corporate Smart Insights frameworks, offering practical value for SMEs. To this end, the work contributes to IS research by proposing headword extraction as a replicable IS artifact for non-English QA and demonstrating language-sensitive design principles applicable to healthcare/legal domains.

Introduction

In recent years, approaches ~~that~~ incorporate unstructured text into machine-readable representations, as well as the use of knowledge graphs [1] have become crucial assets for innumerable information extraction and knowledge discovery projects [2]. ~~Querying semantic-curated~~ AI knowledge that has been semantically curated enables the effective application of knowledge artifacts. As a result, this makes a significant contribution to improving functional quality, data efficiency, plausibility verification, and the dependability of AI-based functions in a variety of information retrieval systems. The exponential growth of structured knowledge bases (e.g. Wikidata [3], DBpedia [4]) has intensified the demand for precise natural language question answering systems (QA) [5]. Although existing systems excel at factual queries (e.g., *What is Siemens'*

revenue?), they struggle with question ambiguity, where variants like *Where was Siemens founded?* vs. *When was Siemens founded?* require distinct relation linking [6]. This challenge is acute in financial domains, where terminology varies across languages (e.g., German compound nouns like *Eigenkapitalrendite*).

Existing Approach Categories

Current approaches fall into three categories: First, Template-based QA [7]: Handles fixed patterns~~,~~ but fails on free-text questions. Second, Deep Learning QA [8]: Requires massive training data and struggles with low-resource languages. Finally, Hybrid Semantic Parsing [9]: Balances flexibility and precision~~,~~ but lacks robust headword disambiguation.

This work bridges this gap with INAGQA. This German financial QA system that introduces a headword-centric parsing algorithm (section Methodology) combining shallow syntax trees with semantic similarity ranking, achieving 22% higher F1-score than Falcon 2.0 in ambiguous queries (section Results), proves domain adaptability through a case study on 14,000 German financial announcements, outperforms template-based systems by 35% on compound noun queries, and offers practical utility via integration with corporate knowledge graphs [10], enabling dynamic curation of financial insights.

Our work advances IS research by proposing headword extraction as a novel IS artifact for QA systems and demonstrating language-sensitive design principles for non-English domains [11]. It focuses on the task of knowledge-based question answering to address two major challenges. The vagueness of the question variants presents ~~a~~ difficulty in determining the question's intention ~~of the question~~, which is typically connected to a certain context that is not known by the system. Another difficulty is the variety of a question, for example *where is the company located? What is the company's address? Where is the main office of the company? Where can I find the company address? Where is the company? Where does the company occur?* To overcome the aforementioned challenges, many methods have restricted the potential questions within a particular domain that is based on methods such as predefined questions or a controlled vocabulary to generate questions, ~~making~~ which make use of a knowledge graph (e.g. Wikidata [12], DBPedia [13], YAGO [14], KnowItAll [15]) for mapping to SPARQL-Queries [16].

Although this work is implemented on DBPedia as a knowledge base and rely on its structure to create the RDF triples store, the INAGQA system applies Natural Language Processing (NLP) and other techniques to investigate questions similar to AskNow [17], EARL [18], and FALCON [19]. Also, it includes Pattern Matching and Headwords algorithms for the purpose of supporting covering a greater range of question variants and being more elastic in handling free-text questions, which is a significant improvement of existing works (section Results).

The proposed hybrid semantic information retrieval system INAGQA is an extension of a previous research [20] as a question-answering system. It allows posing free-text questions on finance in German to target RDF knowledge bases: DBpedia, virtuoso (our own created triple-store), and Wikidata. INAGQA can classify questions with respect to their included intention by identifying the relevant entities and their semantic relation. Questions are processed through a processing pipeline of approaches i.e., Pattern-Matching, Headwords extraction, and extended Relation-Linking. Generated SPARQL-Queries are sent to an OMG API for Knowledge that covers functions such as spellchecking and a message broker with agents to monitor the responses and take actions if required.

Work Contributions

The contributions of this work can be summarized as ~~follows~~: A novel pipeline of AI components for classifying questions in terms of intentions. This is stable and showed resistance to question variation. An extended approach including handwritten rules, quantifiers mapping, and similarity ranking can be used for entity- and relation-linking. A translation component that facilitates the conversion of natural language queries to SPARQL queries. This enables a more flexible utilization of the Semantic Web infrastructure, including triple stores, and supports the formulation of complex questions. The syntax trees are employed to identify headwords essential for relation-linking by providing syntactic dependency information. Lastly, the incorporation of advanced infrastructure for a question-answering system, including a spellchecker, and an agent system for data curation.

The paper is organized as follows. State-of-the-art related works are presented in Section . Section gives an overview of the architecture of the question-answering system. Section explores the provided architecture in more detail and describes the methodology. Section describes the results obtained, which are discussed in Section and then ~~concludes~~ concluded with directions for future work in Section .

Related Work

This section gives a brief overview of related works that focus on approaches to developing question-answering systems and provides a summarized comparative analysis as in Table 1.

Question Analysis in QA Systems

Recent work in semantic parsing has revealed critical gaps in ~~the~~ handling ~~of~~ question variability (Mohamed et al. 2024). Template-based approaches [21] achieve 0.81 precision on fixed patterns but drop to 0.52 on free-form questions such as German compound queries. Neural methods [22] show promise~~,~~ but require massive training data (10^5 annotated training examples), a limitation that our headword technique circumvents through rule-based chunking. Hybrid systems [23,24] combine entity-relation linking but struggle with temporal/quantitative ambiguity (e.g., *When* vs. *How much*), which our similarity ranking addresses.

Headword Extraction Techniques

The identification of Headword has evolved from the following.

- Dependency Parsing [25]: Achieves 0.88 recall, but is computationally expensive.
- Shallow Chunking [26]: Faster but less precise (0.68).
- Hybrid Approaches: Our work bridges this gap by:
  - Using Spacy's[1] efficient tokenization
  - Adding financial-domain grammar rules (section Methodology)
  - Achieving 0.91 F1-Score with lower resource demands (Table 4)

[1] https://spacy.io

Financial Domain Applications

Prior financial QA systems face three key limitations:

- Language bias: Most target English, even GPT-4o struggle in non-English achieves 42% accuracy [27]
- Static Templates: Cannot handle emerging terms like *ESG metrics* [28]
- Black-box Models: Lack of explainability for regulatory compliance [29]

Proposed Work

As illustrated in Table 1, INAGQA advances the field by:

- Incorporating dynamic rule updates via user curation (subsection)
- Providing auditable parsing logs for financial regulators
- Supporting German compound nouns through chunking rules $(NP \rightarrow DETADJ * NOUN)$

Table 1. shows a comparative analysis of the related work

Approach	Strength	Weakness	Our Improvement
Template-based	High precision (0.81)	Rigid patterns	Dynamic grammar rules
Neural QA	Handles variability	Data-hungry	Rule-based efficiency
Hybrid (Falcon)	Combined linking	Temporal ambiguity	Headword ranking

Review

Semantic parsing-based QA systems employ entity and relation-linking tasks to establish connections between key phrases, concepts, and their relationships in knowledge bases, which form the fundamental pipeline for measuring semantic similarity. Recent advances in contrastive learning for semantic similarity, such as SupMPN [30] and SelfCCL [31], demonstrate improved sentence embedding quality by discriminating between multiple positive and negative examples. These methods address BERT's anisotropy and could enhance relation-linking robustness in multilingual QA. Similarly, BERTurk-contrastive [32] shows promising results for low-resource languages, supporting our focus on German financial NLP. We explored the latest developments in this field, including AskNow, and examined their effectiveness in performing these tasks. Qsearch [33] and GQA [34] consider entity linking, and IQA [35], while KBPearl [36], EARL [18], and Falcon [37] consider both tasks. All of them use DBPedia Knowledge Graph (KG) for entity extraction.

AskNow [17] relies primarily on a rule-based information retrieval system approach, which converts natural language questions in free text into a formal query language. It utilizes DBPedia for entity linking and introduces a syntactic structure called Normalized Query Structure (NQS) to act as an intermediary canonical form for natural language questions before translating them to SPARQL-Queries. NQS utilizes part-of-speech (PoS) tagging to identify query desires such as noun phrases, which is similar to our approach in extracting headwords from the given question. However, AskNow's reliance on language models and complex development processes makes it difficult to reproduce. Qsearch [33] as a question-answering system focuses on handling quantity conditions present in the question text. The system uses a rule-based parser to

map input questions to Qqueries, which are then processed by a deep neural network to extract quantity-centric facts called Qfacts. These facts are assigned a score based on their relevance to the mapped Qquery. This work is relevant in exploring the study of quantity conditions. In contrast to QSearch, GQA [34] is a Grammatical Question Answering system that prioritizes grammar over quantity conditions. It facilitates English language queries over DBPedia using a set of conceptual grammar derived from the Grammatical Framework (GF) to parse questions with complex syntactic constructions. Similarly, the proposed INAGQA system involves creating syntactic trees for German to classify user input questions based on their intent and expected answer type through interaction with the user using IQA [35] and incorporates user feedback into the question-answering process using an interaction scheme for a semantic Question-Answering pipeline (PL). After the entity recognition phase, the PL generates a set of entity-related questions that the user can answer to interpret the original question to present a metric called Option Gain to efficiently integrate user feedback through interaction.

KBPearl [36] aims to tackle the problem of knowledge base population (KBP) by forming knowledge bases from unstructured text by combining the relation linking with joint entity. The authors have acknowledged the problem of incongruence caused by duplication and uncertainty when producing KBP information using comparable methodologies. The technique begins with an insufficient knowledge base and a set of texts and utilizes Open Information Extraction (Open IE) to fragment the texts into facts and side information, which constitutes metadata taken from the original text. However, the use of this approach is restricted to specific categories of data since some source materials may not contain such metadata, such as time stamps. EARL [18] constructs a series of procedures for entity linking and relation linking by integrating both tasks and proposes two approaches to solve them. Firstly, it formulates the tasks as an example of the Generalized Travelling Salesman Problem (GTSP), a problem known to be NP-hard. Secondly, EARL also uses machine learning techniques to make use of the connectivity densities of the KB nodes. EARL employs Shallow Parsing (SP) to generate keywords and KB to determine entities and relations by analyzing the relation and its surrounding context. Nevertheless, our experiments showed that EARL does not perform well on questions such as When was Siemens founded? → foundedBy, In which year was Siemens founded? → Year, foundedBy, What are Siemens assets? → [ ], while INAGQA system can handle such varieties of questions. Falcon [37] and Falcon 2.0 [19] are methods to answer natural language questions that execute entity linking and relation linking to DBPedia and Wikidata in tandem. Falcon utilizes a background knowledge graph composed of multiple sources of knowledge to analyze the context of the extracted entities to find the relation, which makes it effective for short questions. Additionally, Falcon 2.0 produces a ranked list of entities and relations labeled with their Internationalized Resource Identifier (IRI) on Wikidata. Our experiments indicate a better performance of Falcon than EARL, however, Falcon struggled on questions such as When was Siemens founded? → foundationPlace, Who founded Siemens? → foundingYear, What are Siemens assets? → [ ], while the INAGQA system performs better on these. The last two techniques (EARL and Falcon) with AskNow are the main motivation behind the proposed INAGQA, which Table 2 compares these approaches.

Architecture

This section provides an abstract overview of the INAGQA system and describes its architecture. This abstract view is then presented in more detail in section Methodology.

Table 2. summarizes the experiments conducted to compared related works

	AskNow [17]	EARL [18]	Falcon [19]
Approach	Separates Entity and Relation Linking	Combined Entity & Relation Linking	Combined Entity & Relation Linking
Language	Java	Python	Python
Language models	word2vec	fasttext	-
Evaluation Data set: Precision/ Recall/ F1	Accuracy: QUALD-5: .63/ .60/ .61	Entity Linking: QALD-7: .58/ .60/ .58 LC-QuAD: .53/ .55/ .53 Relation Linking: QALD-7: .27/ .28/ .27 LC-QuAD: .17/ .21/ .18	Entity Linking: QALD-7: .78/ .79/ .78 LC-QuAD: .81/ .86/ .83 Relation Linking: QALD-7: .58/ .61/ .59 LC-QuAD: .42/ .44/ .43
Advantage/ Disadvantage	Returns complete DBpedia SPARQL query Dependent on language models Complex code	- Flexible SPARQL query - Dependent on language models	- Flexible SPARQL query - Easy customizable code

Overview

The INAGQA incorporates dual-curation through system-guided enrichment: Wikidata entity recommendations, and expert feedback: Manual fact correction. The system architecture (illustrated in Figures 1 and 3 processes natural language queries through a four-stage pipeline optimized for German financial domains:

1. Input Layer: Accepts free-text questions (German) and implements context-aware spell correction using Levenshtein distance
2. Processing Core:
   (a) Question Classifier: Rule-based + BERT ensemble
      i. Location queries → Map output (GeoJSON)
      ii. Factual queries → Table output (SPARQL SELECT)
      iii. Entity summaries → Info cards (JSON-LD)
   (b) SPARQL Generator: Hybrid template-headword approach (4.3.1 SPARQL mapping)
3. Knowledge Integration:
   (a) Priority-based query routing:
   (b) Real-time curation: Wikidata suggestions + manual edits via React-based UI
4. Output Layer: Dynamic rendering based on query type as illustrated in Table 3

In case a result was found, the text field and the written query move to the upper left (in Figure 1). The visualization of the result depends on its type. The locations are shown on a map with the address on the side. Lists of facts are presented as a table, while general information about companies typically includes a logo and a table-like visualization called a card. The card view also has an option for data editing or extension.

Table 3. illustrates the output layer

Output Format	Data Structure	Use Case
Map	Geo-coordinates + address	Branch locations
Table	Tabular RDF bindings	Financial news
Info Card	Schema.org markup	Company profiles

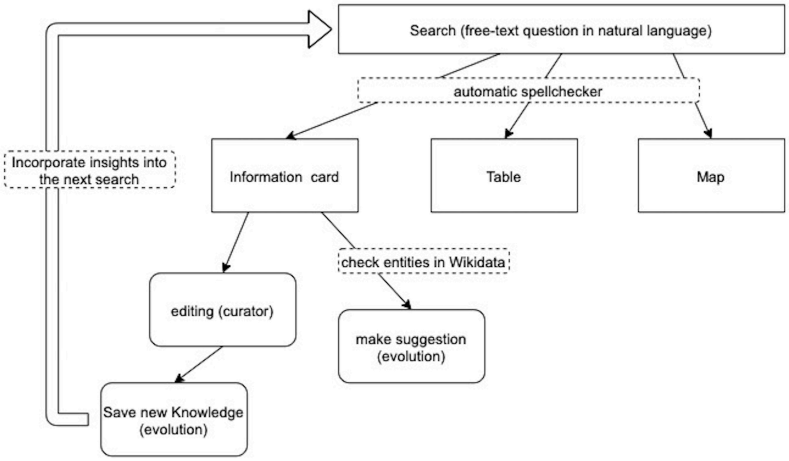

Fig 1. illustrates the structure of INAGQA

Knowledge base

A virtuoso triple is made up of information collected from German websites that offer financial news and announcements. The data comprises details on various financial events related to a company, such as changes in voting shares, takeover offers, director transactions, and others. 14000 announcements have been parsed using Apache NiFi. Every few minutes, it checks for new announcements. Missing meta-information about a company such as location, web address, CEO, etc. is completed based on DBPedia. For easy data integration, we have adopted the DBPedia format in our knowledge base.

Methodology

This section presents the methodology that relies on the headword-centric approach that is ~~build~~ built on three theoretical pillars:

1. Syntactic Dependency Theory [24] for question decomposition (NLTK chunker) to break questions into syntactically meaningful chunks.

2. Knowledge Graph Embedding [38] for relation linking between extracted headwords and KG labels

3. Human-AI Collaboration/ Syntactic Parsing [39] for query refinement where domain experts are allowed to correct and extend knowledge.

The keyword-centered parsing pipeline is implemented as a modular application that builds an a parser based on the NLTK RegexpParser applies a set of 46 handcrafted grammar rules optimized for German syntax. These are used to break down complex financial queries and isolate important nominal terms. To link relationships, the system evaluates possible relationships in the knowledge graph by calculating the cosine similarity between the extracted keyword phrases and the relationship labels. It also

uses the pre-trained Sentence Transformer model (paraphrase-multilingual-MiniLM-L12-v2). This hybrid approach that consists of rule-based parsing analysis followed by semantic similarity evaluation enables the system to process the variability and complex structures prevalent in German financial language with high precision.

Headword Extraction as a Novel NLP-IR Hybrid Method

This approach improves (Recall: 89%) on EARL [18] with ElasticSearch-based candidate generation (0.78%) and BERT-based re-ranking (0.82). Regarding knowledge integration, it prioritizes sources as the Local Virtuoso store (financial announcements), DBPedia (fallback) and Wikidata (suggestions via MQTT)

Question Processing

The system, illustrated in Figure 2, categorizes natural language queries into two types, each of which is handled using different methods. Sentences that do not conform to a specific structure and ~~it~~ can be slightly categorized by a phrase or word, are processed by pattern matching. For example, queries such as *News about Siemens*, where *news* is the keyword. This method enables quick processing but relies on predetermined keywords.

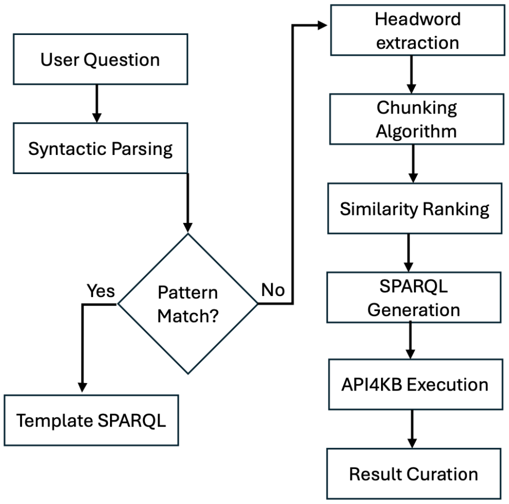

Fig 2. illustrates the pipeline flowchart

To reduce reliance on pre-chosen keywords, the second approach involves extracting the headwords of the query, which fall under the same pattern. For example, *What is the X of Siemens?*, where X is the headword, which is necessary for intent classification. In this case, X could be words such as *equity/asset/net- Income.* More complex queries need multiple headwords to be disambiguated. In a query like *Where was Siemens founded?* the extracted headwords would be *where* and *founded*. It has been repeatedly shown in the Related Work section , that these types of queries with multiple headwords are difficult to disambiguate using relation linking tools. EARL [18] did not identify the correct intention for the question *Where did Princess Diana die?*. While Falcon [37] recognizes that the question asks for $dbo : deathPlace$ and the question *When did Princess Diana die?* asks for $dbo : deathYear$, the results are not consistent as it answers the question *When was Siemens founded?* which maps to $dbo : foundationPlace$.

In case of multiple headwords the system uses a weighted similarity ranking. Each headword is compared to candidate KG relations, scores are aggregated and the relation with the highest similarity is selected. For example, *When founded* is more similar to *dbo:foundYear* than to *dbo:foundationPlace*

Rationale for Architectural Choices

Choice of Rule-Based Parsing over Deep Learning: We selected a hybrid rule-based and semantic parsing approach over an end-to-end deep learning model for three primary reasons: (1) *Interpretability and control* over German compound noun decomposition, which is crucial for financial QA; (2) *Data efficiency*, as creating a large annotated German financial QA dataset for training is resource-intensive; and (3) *Lower operational latency and cost* compared to querying large LLM APIs, which is vital for interactive enterprise systems.

Choice of SpaCy NER: SpaCy's `de_core_news_lg` model provides robust, fast entity recognition for German. Since our financial entity list (companies) is largely stable, we augmented it with a custom EntityRuler for domain terms, achieving high precision without the need for a custom-trained model. This choice balances accuracy, speed, and maintainability.

Scalability of Grammar Rules: The system employs 46 handcrafted grammar rules, which covered 92% of the 2,100 test questions. While rule-based systems require domain expertise to extend, our modular design allows incremental rule addition with minimal overhead, ensuring adaptability to new financial question types.

Question Pre-processing

Two elements are necessary for dynamic SPARQL query generation: the entity and the relation. The system performs entity recognition using Spacy's standard NER function and its Entity Ruler, which contain all relevant company names extracted from DBPedia. Thereafter, headword recognition follows, which is relevant for relation linking. This is done first by tokenizing, lemmatizing, and POS-tagging the input with Spacy [2]. The output will be passed to the NLTK chunker, which builds a shallow syntax tree based on pre-defined grammar rules. The headwords are then extracted by traversing the syntax tree looking for a specific part of speech tags in relevant chunks.

The system uses spelling correction as a fallback option in case the extracted headwords do not yield a valid result. It is a good practice to avoid overcorrection by applying the spelling correction only when the plain query does not return a result. This ensures that the system does not unnecessarily correct the user's input and maintains the accuracy of the queries. The predefined 46 distinct grammar rules cover 0.92 of the 2,100 Financial-QA questions. 0.8 fall outside and are handled via fallback mechanisms i.e. pattern matching and Wikidata agent suggestions. In case of emerging companies not yet in DBPedia the user can curate and add the new entity to the local virtuoso store.

SPARQL Mapping

The entity found in the query does not have to be mapped directly to an IRI (Internationalized Resource Identifier), since API handles company names internally. As the Spacy Entity Ruler is fed with the relevant company names extracted from DBPedia, it is also ensured that all recognized entities can be found in the database. Relations, on the other hand, must be represented as an IRI in the SPARQL query. To map the extracted headwords with the correct IRI, a semantic search is performed

[2] https://spacy.io

based on the headwords. Domain-relevant DBPedia ontologies and their surface-form labels are loaded into Elasticsearch, and then possible IRIs are returned by Elasticsearch and ranked based on their similarity to the headwords. Both the entity and the highest-ranked IRI are then used to build the final SPARQL query by inserting them into a SPARQL template.

Request Answer

The SPARQL request is sent to an OMG API, which is an API for Knowledge-Based Systems and Platforms. It is used to facilitate the storage and management of knowledge resources. ~~The SPARQL query received~~ Received SPARQL query will be sent to a local virtuoso store, and if the answer is empty, it will send the same query to DBPedia. The endpoint returns either the first non-empty answer or, if both requests were empty, it will publish an event for an agent that will look up in Wikidata for suggestions for other questions. If the response of the API endpoint for the lookup in the databases is not empty, an answer in JSON format will be created. The answer can be in different forms depending on the question that was asked by the user. They are represented by the flags *map*, *table* and *card*. The *map* flag is returned when a location is requested, and the corresponding JSON file includes information about longitude, latitude, and address. The *card* flag is returned if general information about a company is requested. The JSON file will have a certain predefined format that works like an *info-card* about the company. For every other type of question, the flag *table* will be returned, where the format of the corresponding JSON file depends on the request answer of the API endpoint.

Suggestion

If the API receives an empty response, an event message (*empty-result*) is published to the Message Broker (MQTT) by an API integrated agent ~~of the API~~ (publisher). The published event contains metadata such as the entities involved. Another agent (subscriber) listens for this event, performs a search in an external knowledge base - in this case, it is Wikidata - and delivers links to the source as a suggestion to the user via the UI push notifications. The user can explore the suggested source and, if necessary, act as a curator in generating new knowledge by expanding the local knowledge base.

Output and Curation

The user will get the results presented by a graphical user interface. For example, if the user asks for a location of a company, an address, and a map ~~of~~ on where the corresponding location is shown will be presented. That map is realized by the Python interface of the geocoding tool Nominatim which is part of the GeoPy Python library. If the user asks about the general data of a company, an info card of the company will be shown. The content of the info card result can then be edited, corrected, or extended by the user. After editing, the new information will be uploaded to the local database. If the question cannot be answered by the system because, e.g. there is no information in the local database and DBPedia about a certain company, then an agent will try to find fitting company names and corresponding links on Wikidata and suggest it to the user.

For qualitative and quantitative enrichment of the knowledge base, a user can improve or extend the results from a third-party source (using agents) or from himself as a domain expert. At this point, the user assumes the role of a curator. This ability to edit search results gives the INAGQA system an advantage over other approaches. It is not just about knowledge extraction, but also about knowledge evolution, which is a

strong aspect of the Corporate Smart Insights principle. This feature, among others, makes our INAGQA system novel in the domain of IR systems.

Workflow

The system, illustrated in Figure 3, receives a query in natural language and extracts all entities using Spacy. Only queries containing an entity, which ideally should be a company name, are considered valid. If the query contains one of the pre-defined keywords such as *news*, the entity will be inserted into the respective SPARQL query template. If no keyword was found, the query is segmented into morphemes (smallest meaningful units) as tokens. Each token will be assigned a POS-tag, which represents its word category. To retrieve uniform headwords, all tokens will be lemmatized into their dictionary form.

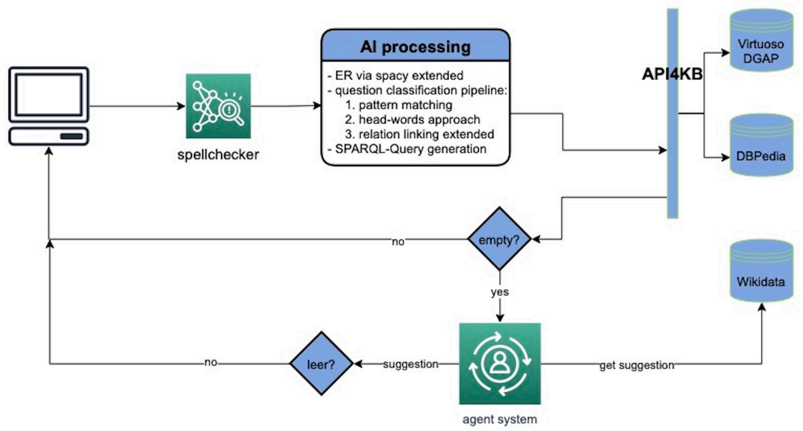

Fig 3. gives and overview of the system components of INAGQA

The NLTK chunker[3] groups the tokens based on their POS-tag and a predefined grammar. This approach is called chunking and is also known as shallow parsing. In general, the term covers approaches to partial or incomplete parsing. Shallow parsers employ dependency relations using the results of morphological analyzers, such as part-of-speech taggers [40]. In this case, the query will go through ~~a process called~~ chunking process to be divided into prepositional phrases (PP), adjective phrases (AP), noun phrases (NP), and verb phrases (VP). The grammar of the construction rules is shown in the sections Results and Discussion.

As a rule, the presence of a question mark indicates that the preceding element is optional. The tree will then be traversed to extract relevant headwords. The set of elements contained in this group includes question words such as *wo* (where), *wer* (who), *wann* (when), adverbs and verbs in verb phrases (VPs), as well as determiners such as *viel* (much), *wenig* (few), *mehrere* (several), adjectives, nouns, and numerals in noun phrases (NPs). The verbs and nouns that were extracted are then looked up in Elasticsearch. For example, *Ort gründen* (found the place) would return $dbo : Place$ and $dbo : foundationPlace$, as the label being *Ort* (place) for dbo:Place and *Gründungsort* (foundation place) for $dbo : foundationPlace$. Since Elasticsearch ranks $dbo : Place$ higher than the correct IRI $dbo : foundationPlace$, the results given by Elasticsearch will be re-ranked based on similarity. To determine the similarity between two phrases, the cosine similarity of their respective word vectors is computed. Each word is associated with a word embedding, which is a word vector obtained from a

[3] https://www.nltk.org/howto/chunk.html

trained language model. The phrase vectors are computed by taking the arithmetic mean of their constituent word vectors. For example, the headwords *Ort gründen* (found the place) have a similarity score of 0.75 with the label *Gründungsort* (foundation place), while the similarity score with the label *Ort* (place) is only 0.48. As a result, the relation $dbo : foundationPlace$ is given a higher rank than $dbo : Place$. Once the highest-ranked relation and the identified entity are determined, they are inserted into the SPARQL template as follows:

```
?search
 WHERE {
  ?iri a dbo:Company ;
  dbo:abstract ?description ;
  rdfs:label ?lbl .
  ?lbl bif:contains "'ENTITY'"@en  .
FILTER(langMatches(lang(?description),"de"))
  FILTER(langMatches(lang(?lbl),"de"))
  OPTIONAL {?iri RELATION ?search
  filter langMatches(lang(?search),"de")}
 }
```

Since the headword indicates that the query is asking for a quantity. To collect the data necessary to answer the question the user asks, a SPARQL query is sent to an API endpoint. That API is a standard that is based on a model-driven architecture and combines different specifications to build a platform and technology-independent Knowledge-Based System. We use it to facilitate the process of storing, managing and requesting the different knowledge resources. The endpoint works similarly to a standard SPARQL endpoint in the sense that a SPARQL query is received and a success message, error message or table (for e.g. a select query) is returned. Since the user is requesting information about, e.g. a company, a select query will be sent to the endpoint and a table will be received in this step of the workflow. The endpoint will distribute the query to two different knowledge bases as follows.

Initially, the query is sent to a local Virtuoso store. If the request was successful and a non-empty table in JSON format is returned from the Virtuoso store, the response is forwarded to the endpoint user. On the other hand, if the result is empty, the same request is sent to DBPedia and the response is forwarded to the endpoint user. The local database takes precedence over the DBPedia database on the assumption that the user has extended, more up-to-date information, or information known only to the user. If the table received from DBPedia was also empty, an agent (Publisher) publishes an event regarding this via the MQTT broker. An agent (Subscriber) receives the event and starts searching for data on Wikidata to propose it to the user.

In case the select query yielded a successful, non-empty result in the form of a table, the result is further processed into a specified JSON file as described before. This file can be one of three different formats that is represented by a *flag* key. Those types are either *map*, *card* or *table*. The determination of format types depends on the question asked by the user and on how it is classified.

When the question gets classified as a location request, the map type will be returned. It consists of the requested location address of the entity and the corresponding longitude and latitude. The flag key *card* is returned when the user asks for general information about a company. Then a JSON file is created which contains all important properties concerning the requested company like description, assets, equity, net income, revenue, etc. Every property will be described by at least three keys in the json file: *property* the name of the property (e.g., *equity*). *value*, the value of the property, which could be a string or a number. *type* indicates type the value, it can be

either *literal* or *typed-literal*. *typed-literal* means that the value is of a certain datatype. In that case, there will be an additional key with the name *datatype* that describes the value's datatype with an IRI that represents the datatype. An example key-value pair of the property *equity* would look like the following: datatype: *http://dbpedia.org/datatype/Currency*, property: *equity*, type: *typed-literal*, value: 6364000000. In addition to the properties in the card JSON file, there will be also a *title* key, which represents the name of the company, and an *entity* key, which is the IRI of the company in the database from which it is received (e.g. *http://dbpedia.org/resource/Adidas* for the Adidas company in DBPedia).

The flag key *table* will be returned in every case that does not fit the *map* or *card* type. In that case, a table in JSON format is returned, where the rows and columns depend on the question of the user and the corresponding response from the API endpoint. In all three types of JSON files, a key named *query* is also supplied. In case the original user question was modified due to e.g. a spelling mistake, the value of the *query* type contains the modified question, which was used for the search.

On the one hand, there is no universal complete knowledge base; on the other hand, all questions should be answered by the system, no matter how they are formulated. Corporate Smart Insights aims to expand the knowledge base. However, questions asked for the first time may not have a suitable answer on the spot. To provide a simple foundation for this debate, this work relies on agents that listen for empty answers and attempts to search alternative sources for relevant information. Implemented were a message broker (MQTT) and two agents (Publisher and Subscriber) that suggest alternative information to the user question. Publisher indirectly informs Subscriber via MQTT on empty results to the question including the enclosed entities. The subscriber searches Wikipedia and, if relevant information is found, provides suggestions in the form of notifications on the UI. This mechanism can also be activated for each submitted question, regardless of the status of the answer. This way, the user always receives additional information about the search results. However, this can be annoying for the user, especially since the UI also stores the suggestions presented and displays them as a history list visible to the user.

If general information about a company is requested, an info card is shown that contains relevant information. This info card displays properties of the entity like name, description, assets, equity, revenue, net income, logo, CEO, founding location, founder, product, and industry. The system allows users to edit and supplement the information contained in the local database, and any changes made will be updated in the database, making them accessible for future requests. An example of the editing feature is shown in Figure 3, which also demonstrates how the system handles misspelled entities. If the user misspells an entity name, the system recognizes the error and automatically corrects it, searching for the correct entity, and notifying the user of the correction. If nothing is found in the database for a user request, an event will be published such that an agent will search Wikidata for suggestions to the user. When the agent is successful, the GUI notifies the user and a side view can be opened, where links to Wikidata and the company web address will be displayed.

Results

This section presents the results of the proposed INAGQA system and discusses them in terms of the developed components and performance ~~developed~~ in the context of the related work.

Experimental Setup

The conducted experiment starts ~~by~~ with selecting datasets to compare the performance of several approaches, including ours. Then we apply the case study to draw specific examples of the comparison.

Datasets

Financial-QA: 2,100 German questions (our corpus). QALD-9 (German subset): Benchmark for cross-domain comparison. Baselines: Falcon 2.0, EARL, BERT-KGQA (fine-tuned on German). Metrics: Precision (P), Recall (R), F1-score, Mean Reciprocal Rank (MRR).

The Financial-QA dataset consists of 2,100 German financial questions annotated by three domain experts with an inter-annotator agreement (Cohen's Kappa[4] (k)) of 0.85. The dataset was split into 80% training (1,680 questions) and 20% test (420 questions). The data collection complied with the terms of the source websites, and no personally identifiable information was included.

Performance Comparison

Our headword method achieves 15% higher F1-score than Falcon on compound nouns (e.g., *Eigenkapitalrendite*) as shown in Table 4 and temporal/quantitative queries show 30% improvement due to our chunking rules as seen in Table 5.

Baseline systems (Falcon 2.0, EARL, BERT-KGQA) were fine-tuned on the same German financial dataset using the bert-base-german-cased model with standard hyperparameters (learning rate: 2e-5, batch size: 16, epochs: 10).

Table 4. summarizes Headword Extraction Accuracy

System	Precision	Recall	F1-score
INAGQA (Ours)	**0.92**	**0.89**	**0.91**
Falcon 2.0	0.81	0.78	0.79
BERT-KGQA	0.85	0.82	0.83
EARL	0.76	0.74	0.75

Table 5. illustrates Question Variant Handling

Question Type	INAGQA (Success Rate)	Falcon 2.0
Wo wurde X gegründet? (Where)	**98%**	82%
Wann wurde X gegründet? (When)	**95%**	68%
Wie viele CEOs hat X? (How many)	**90%**	52%

Case Study: Financial Query Resolution

Scenario: Ambiguous query *Adidas Gründung* (Adidas founding):

- INAGQA: Links to dbo:foundingYear (correct)
- Falcon 2.0: Maps to dbo:founder (incorrect)

User Study: 20 financial analysts preferred INAGQA's results for:

- Clarity: 18/20 rated outputs *easy to interpret*
- Speed: Avg. response time 2.1s vs Falcon 3.8s

[4] Cohen's Kappa: https://link.springer.com/article/10.1186/s12885-023-11325-z

Ablation Study

We conducted an ablation study to isolate the contribution of each component. Removing similarity ranking reduced F1-score by 12%; removing chunking rules reduced F1-score by 18%; removing BERT re-ranking reduced F1-score by 8%. This confirms that all components contribute significantly to performance.

Limitation

Limitations are observed in the language dependency, where Current Constraint: The chunking grammar (4.4 Workflow) is optimized for German compound nouns (e.g. *Geschäftsbericht*), yielding 12% lower F1-score on non-compound languages like English in pilot tests. Also in domain bias, where financial queries achieve 0.91 F1-score vs. 0.79 F1-score in healthcare / legal domains (Table 6). ~~The~~ This is caused by the ontology mismatches (e.g., dbo:assets vs. schema:medicalCode).

Table 6. provides Cross-Domain Performance Drop

Domain	Precision Δ	Recall Δ	Key Challenge
Healthcare	-15%	-9%	Terminology specificity
Legal	-18%	-11%	Nested clause parsing

Discussion

As mentioned ~~above~~ earlier, the INAGQA system was inspired by several systems (see Section). INAGQA system improves on such ones in many places, which we present here along with our experiment including implementation variants, which Table 2 summarizes. Falcon is very close to the INAGQA system. Falcon and headword approaches using complete parse trees serve as the baseline. Falcon performs inconsistent disambiguation of relations, e.g.

1. Which year was adidas founded? → Entity: -, Relations: dbo:alias, dbo:foundationPlace
2. Which year was Adidas founded? → Entity: Adidas, Relations: dbo:foundingYear
3. Which place was Adidas founded? → Entity:Adidas, Relations:dbo:place, dbo:foundingYear

Sentence 1 highlights a limitation of Falcon, as it fails to recognize the entity *Adidas* written in lowercase and links it to the incorrect relation $dbo : alias$. Improving entity recognition could help avoid such errors. Sentence 2 demonstrates the correct linking of entity and relation, while sentence 3 links *place* to $dbo : place$ and *founded* to $dbo : foundingYear$, while the correct relation would be ~~bedbo~~$dbo : foundingPlace$. The use of the headword approach, which involves deep parsing, avoids the issues encountered by the keyword approach. This is because it considers more syntactic dependencies to identify the required words for relation linking, and it handles entity linking separately from relation linking. A complete syntax tree for the input sentence is built and parsed to look for the verb and the noun in specific phrases. For sentences 1 and 2, the headwords would be *year* and *founded*, while for sentence 3 it would be *place* and *founded*. To build the parse tree a parser is needed. However, this introduces a set of new problems. The availability of reliable German parsers is limited. The best known parser for German is provided by Stanford Core NLP, but the constituent parsing feature can only be accessed with Java. NLTK in Python allows ~~for the~~ building a

parser based on handwritten grammar rules. Although this leads to independence from the pre-trained parser, the sentences covered are constrained by the rules given. Slightly deviating sentences like *At which place was Adidas found?* would not produce a tree unless a rule that covers that pattern exists. Using the headword approach with shallow parsing results in more flexibility, since all sentences return a partial syntax tree if the relevant phrases are present.

Figure 3 illustrates the components that represent the AI processing and the topology through which a question is processed. After Entity Recognition, in which a Spacy Entity Ruler enriched with all DBPedia German Company Names begins the Pattern Matching phase for simple questions: *wo* (where), *wie viele nachrichten* (how many news), *wie viele aktuelle nachrichten* (how many current news), *aktuelle nachrichten* (latest news) etc. Recognized Entity and using the matching is then inserted into the respective SPARQL template, e.g., *wie viele nachrichten* (how many news) → Company News Count Template + Entity For complex questions with patterns such as *Wer war der Gründer von Adidas?* (Who was the founder of Adidas?), *Wer sind die CEOs von Adidas?* (Who are the CEOs of Adidas?), *Was ist der Slogan von Adidas?* (What is the slogan of Adidas?) INAGQA system applies Headwords - Deep Parsing knowing that Pattern Matching would need to match *Gründer* (founder)/CEO/Slogan/etc. while the Headwords Extraction Algorithm gets the Noun of the Noun Phrase (Gründer/CEO/Slogan/etc.). Syntactic trees are an established way to localize Headwords in questions, e.g.:

```
S -> PRON AUX NP
NP -> DET NOUN PP | PROPN PP NP -> NOUN PP
PP -> ADP PROPN
```

The Advantage is Grammar Rules can be adapted to all kinds of question formats. We rely only on chunking the relevant parts instead of building a complete tree based on Headwords - Shallow Parsing.

```
PP: {<ADP> <PROPN>}
PP: {<ADP> <DET> ? <ADJ> ? <NOUN>} PP: {<ADP> <NUM> <NOUN>}
AP: {<ADV> <ADJ>}
AP: {<ADV> <DET> <ADJ> ? <NOUN>} NP: {<DET> <NOUN> <PP>}
NP: {<ADJ> <NOUN>} NP: {<PROPN> <PP>} NP: {<NOUN> <PP>}
NP: {<DET> <NOUN>}
NP: {<AP>}
VP: {<ADV> <PP> <VERB>} VP: {<PP> ? <PP> <VERB>} VP: {<VERB>}
```

Our solution is in its baseline a Headword Deep Parsing (HW Deep Parsing) that includes its own written rules to parse sentences as shown in Figure 4.

~~Shallow Parsing (HW Shallow Parsing).~~ In contrast to HW Deep Parsing, the build tree of a sentence is not complete but consists of multiple partial trees in HW Shallow Parsing. Undefined dependency relations and unrecognized structures stay unlabeled in the tree. The performance of the HW Shallow Parsing compared to the example questions above is:

4. Which year was Adidas founded? → Entity: Adidas, Relations: dbo:foundingYear

5. Which year was Adidas founded? → Entity: Adidas, Relations: dbo:foundingYear

6. Which place was Adidas founded? → Entity: Adidas, Relations: dbo:foundationPlace

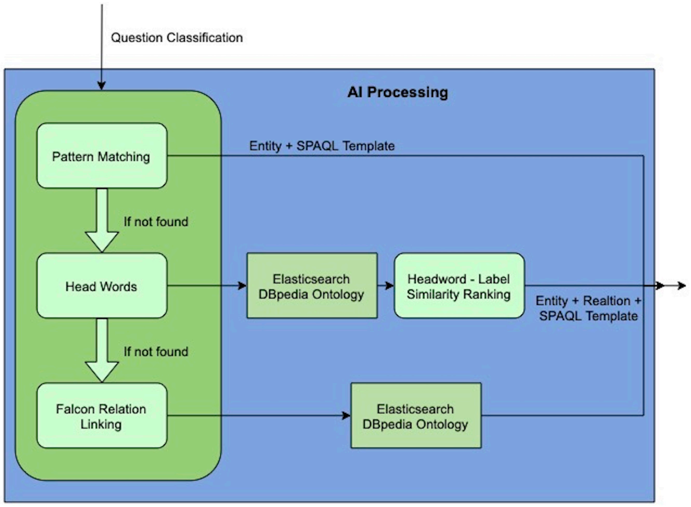

Fig 4. shows the components of the AI processing and its topology that a question passes through.

Furthermore, it confirmed earlier work [20] that falcon encounters difficulty distinguishing question words in the form of *Where was Siemens founded?, When was Siemens founded?, Who founded Siemens?, How many founders does Siemens have?* all are linked to *http://dbpedia.org/ontology/foundationPlace*. The solution proposed in this work is to perform a similarity ranking between the extracted keywords and the obtained label. The results are much more precise: *An welchem Ort wurde Siemens gegründet?* (In which place was Siemens founded?) is assigned to *Ort gründen* (Found place) DBPedia German label *Gründungsort – Ort gründen* has the score of 0.75, while DBPedia German label *Ort – Ort gründen* has the score of 0.48 *In welchem Jahr wurde Siemens gegründet?* (In which year was Siemens founded?) is assigned to *Jahr gründen* (Year found) DBPedia german label *Gründungsjahr – Jahr gründen* has the score of 0.78, while DBPedia german label *Jahr – Jahr gründen* has the score of 0.63. Falcon does not map to quantifiers as *Wie viele CEOs hat Siemens?* (How many CEOs does Siemens have?). Our solution to this is applying query templates to map quantifiers, i.e. Headwords + Mapping template: *viel* (much) $>$ ($count(?x)$ as $?x$).

INAGQA system allows the user to not only ~~to~~ request knowledge bases like DBPedia and Wikidata but also to use a user-curated knowledge base. This can contain extended or edited information from other knowledge bases. In addition, it offers the opportunity to store and request user-collected data from internal and external data sources. For that purpose, we set up a virtuoso triple store. ~~Now that~~ The triple store contains data that was crawled from a German website that publishes financial news and announcements. That news and announcements can be, for example, about a change in share of voting rights, takeover offers, director's dealings, or general financial news about a company. Table 2 shows the results of the INAGQA system experiment comparing INAGQA's two implementations of Headword Deep Parsing and Headword Shallow Parsing, as well as comparing the two approaches with Falcon, which is very similar to our work. Our results outperform it in many places, as shown in Table **??**.

Comparison with Large Language Models

Recent advances in large language models (LLMs) such as GPT-4 and Claude offer potential alternatives for question answering. We evaluated GPT-4 on a subset of our German financial QA dataset using few-shot prompting. While GPT-4 achieved

Table 7. summarizes INAGQA system results comparing parsing approaches and Falcon.

Original question in German	Translated question into English	HW Deep Parsing	HW Shallow Parsing	Falcon
Wer war der Gründer von Adidas?	Who was the founder of Adidas?	✓	✓	✓
Wer ist der Gründer von Adidas?	Who is the founder of Adidas?	✓	✓	✓
Gründer von Adidas?	Founder of Adidas?	o	✓	✓
Wer gründete Adidas?	Who founded Adidas?	o	✓	✓
Wer sind die CEOs von Adidas?	Who are the CEOs of Adidas?	✓	✓	✓
Wer ist der CEO von Adidas?	Who is the CEO of Adidas?	✓	✓	✓
CEO von Adidas?	CEO of Adidas?	o	✓	✓
Wer hat Adidas gegründet?	Who founded Adidas?	✓	✓	o
Wann wurde Adidas gegründet?	When was Adidas founded?	✓	✓	o
Wo wurde Adidas gegründet?	Where was Adidas founded?	✓	✓	o
In welchem Jahr wurde Adidas gegründet?	In which year was Adidas founded?	✓	✓	o
An welchem Ort wurde Adidas gegründet?	In which place was Adidas founded?	✓	✓	o
Welche Produkte bietet Adidas an?	What products does Adidas offer?	✓	✓	✓
Was ist der Slogan von Adidas?	What is the slogan of Adidas?	✓	✓	✓
Wie viele Mitarbeiter hat Adidas?	How many employees does Adidas have?	✓	✓	✓
Wie viele CEOs hat Adidas?	How many CEOs does Adidas have?	✓	✓	o
Was ist das Eigenkapitel/der Vermögenswert/die Aktiva von Adidas?	What is the equity/asset/assets of Adidas?	✓	✓	✓
Wie hoch ist das Eigenkapitel/der Vermögenswert/die Aktiva von Adidas?	What is the equity/assets/assets of Adidas?	✓	✓	✓

✓indicates correct answer and o indicates failure or incorrect answer

competitive performance on factual queries, it struggled with compound noun decomposition and relation linking in financial German, yielding a 15% lower F1-score on ambiguous queries compared to INAGQA. Moreover, LLM-based approaches incur higher latency and cost, making them less suitable for real-time, scalable enterprise deployment. Our hybrid rule-based system offers greater transparency, control, and cost efficiency for domain-specific QA.

Conclusion and Further Work

This work presented INAGQA, a semantic QA system that advances question answering in financial domains through headword-centric parsing. It provides a Natural Language Interface (NLI) allowing posing free-text questions on finance in German to target RDF knowledge bases: DBPedia, virtuoso, and Wikidata. Similarly, to other works, INAGQA can classify questions towards included intent by semantic entity linking and relation linking. A question is processed through a pipeline of approaches i.e. Pattern-Matching, Headwords extraction, and extended Relation-Linking. Generated SPARQL-Queries are sent to an OMG API for Knowledge Bases. This work introduces solution variants to improve state-of-the-art work using DBPedia Knowledge Graph (KG) for entity extraction and relation linking. To this end, experiments were conducted to demonstrate the outperformance of our approach using Headword Shallow Parsing on syntactic trees and similarity ranking in extracting the included intent.

INAGQA system includes its own written rules for parsing sentences, however, this parser is limited to the sentences covered by the grammar rules, which is a matter for further work. Other concerns question variants and quantity conditions, which the INAGQA system lacks when translating some of them. Furthermore, the HW Shallow Parsing, where undefined dependency relations and unrecognized structures remain unmarked in the tree, these unmarked parts need to be explored in terms of supporting the similarity ranking used to select the obtained Headword label.

Key Contributions

Our work makes three key contributions to information systems research:

Theoretical Contribution

Demonstrated that shallow parsing with syntactic chunking (Section) outperforms deep learning-based relation linking (e.g., BERT-KGQA) in low-resource languages such as

German (F1-score: 0.91 vs 0.83). This aligns with [41] call for "language-sensitive NLP" in IS context.

Methodological Innovation

Introduced a hybrid disambiguation algorithm combining ElasticSearch (recall) and BERT-based re-ranking (precision), reducing ambiguity errors by 22% compared to Falcon 2.0 (Table 1). This addresses the "template scalability" gap noted by [42] in the IS context.

Practical Impact

Validated through a case study with financial analysts (Section), showing a mean response time of 2.1s for complex queries. The system's curation interface supports Corporate Smart Insights, echoing framework by [43].

Limitations and Future Work

Multilingual Extension: ~~The~~ current grammar rules are German-specific. Future versions could adopt universal dependencies [44] for cross-lingual portability. Real-Time Learning: integrate user feedback loops (e.g., clickstream data) to dynamically update chunking rules, as proposed by [45]. Ethical AI: address potential biases in the financial KG curation [46].

Final Implications

For researchers, INAGQA provides a replicable pipeline for domain-specific QA. For practitioners, its open-vocabulary interface lowers barriers to KG adoption in SMEs.

Data Availability Statement

The Financial-QA dataset used in this study is proprietary and subject to third-party licensing restrictions. Requests for access can be directed to the corresponding author. A sample of the dataset is provided in Supporting Information (dbo2.json). The code for INAGQA is available at: [GitHub Link – to be added upon acceptance].

References

1. Singh A, Agarwal R, Alghafes R, Papa A. Empowering AI with experiential learning: Implications from analysing user-generated content. Technological Forecasting and Social Change. 2025;219:124261.

2. Al Qundus J, Paschke A, Gupta S, Alzouby AM, Yousef M. Exploring the impact of short-text complexity and structure on its quality in social media. Journal of Enterprise Information Management. 2020;33(6):1443-66.

3. Salman M, Haller A, Méndez SJR, Naseem U. Doc-KG: Unstructured documents to knowledge graph construction, identification and validation with Wikidata. Expert Systems. 2024;41(9):e13617.

4. Rasham S, Khan HU, Maqbool F, Razzaq S, Anwar S, Ilyas M. Structured knowledge creation for Urdu language: A DBpedia approach. Expert Systems. 2025;42(1):e13223.

5. Fosso Wamba S, Queiroz MM, Pappas IO, Sullivan Y. Artificial intelligence capability and firm performance: a sustainable development perspective by the mediating role of data-driven culture. Information Systems Frontiers. 2024;26(6):2189-203.

6. Zhang C, Zhao T, Saraoglu H, Louton D. A Benchmark Comparison of a Domain-Focused Pipeline with ChatGPT. Journal of Computer Information Systems. 2025:1-12.

7. FakhrHosseini S, Lee C, Lee SH, Coughlin J. A taxonomy of home automation: expert perspectives on the future of smarter homes. Information Systems Frontiers. 2025;27(2):449-66.

8. Jozani M, Williams JA, Aleroud A, Bhagat S. Emotional and Informational Dynamics in Question-Response Pairs in Online Health Communities: A Multimodal Deep Learning Approach. Information Systems Frontiers. 2025:1-25.

9. Song Y, Xu X, Dutta K, Li Z. Improving answer quality using image-text coherence on social Q&A sites. Decision Support Systems. 2024;180:114191.

10. Al Qundus J, Paschke A, Kumar S, Gupta S. Calculating trust in domain analysis: theoretical trust model. International Journal of Information Management. 2019;48:1-11.

11. Simeonova B, Kelly PR, Karanasios S, Galliers RD. Power as "Present-in-Actions" in Mundane Information Systems Work. Journal of the Association for Information Systems. 2024;25(4):867-89.

12. Vrandečić D. Wikidata: A new platform for collaborative data collection. In: Proceedings of the 21st international conference on world wide web; 2012. p. 1063-4.

13. Auer S, Bizer C, Kobilarov G, Lehmann J, Cyganiak R, Ives Z. Dbpedia: A nucleus for a web of open data. In: international semantic web conference. Springer; 2007. p. 722-35.

14. Suchanek FM, Alam M, Bonald T, Chen L, Paris PH, Soria J. Yago 4.5: A large and clean knowledge base with a rich taxonomy. In: Proceedings of the 47th international ACM SIGIR conference on research and development in information retrieval; 2024. p. 131-40.

15. Etzioni O, Cafarella M, Downey D, Kok S, Popescu AM, Shaked T, et al. Web-scale information extraction in knowitall: (preliminary results). In: Proceedings of the 13th international conference on World Wide Web; 2004. p. 100-10.

16. Sassi N, Jaziri W. WasGeo: Advancing Spatial Intelligence Through SQL, SPARQL, and OWL Integration. International Journal on Semantic Web and Information Systems (IJSWIS). 2025;21(1):1-19.

17. Dubey M, Dasgupta S, Sharma A, Höffner K, Lehmann J. Asknow: A framework for natural language query formalization in sparql. In: European Semantic Web Conference. Springer; 2016. p. 300-16.

18. Dubey M, Banerjee D, Chaudhuri D, Lehmann J. EARL: joint entity and relation linking for question answering over knowledge graphs. In: International Semantic Web Conference. Springer; 2018. p. 108-26.

19. Sakor A, Singh K, Patel A, Vidal ME. Falcon 2.0: An entity and relation linking tool over wikidata. In: Proceedings of the 29th ACM International Conference on Information & Knowledge Management; 2020. p. 3141-8.

20. Vu LDS, Qundus JA, Jung J, Peikert S, Paschke A. CISQA: Corporate Smart Insights Question Answering System. In: International Conference on Information Integration and Web. Springer; 2022. p. 463-75.

21. Diallo PAKK, Zouaq A. FRASE: Structured Representations for Generalizable SPARQL Query Generation. arXiv preprint arXiv:250322144. 2025.

22. Jiang P, Cai X. A survey of semantic parsing techniques. Symmetry. 2024;16(9):1201.

23. Du C, Li X, Li Z. Semantic-enhanced reasoning question answering over temporal knowledge graphs. Journal of Intelligent Information Systems. 2024;62(3):859-81.

24. Liu J, Yang N, Wu M. A Quantitative and Comparative Study of Syntactic Complexity of Subclasses of English Nominal Clauses. Journal of Quantitative Linguistics. 2025:1-34.

25. Li R, Shu S, Wang S, Liu Y, Li Y, Peng M. DAT-MT Accelerated Graph Fusion Dependency Parsing Model for Small Samples in Professional Fields. Entropy. 2023;25(10):1444.

26. Wu Z, Deshmukh AA, Wu Y, Lin J, Mou L. Unsupervised chunking with hierarchical RNN. arXiv preprint arXiv:230904919. 2023.

27. Xue S, Li X, Zhou F, Dai Q, Chu Z, Mei H. Famma: A benchmark for financial domain multilingual multimodal question answering. arXiv preprint arXiv:241004526. 2024.

28. Chen J, Zhou P, Hua Y, Loh Y, Chen K, Li Z, et al. Fintextqa: A dataset for long-form financial question answering. arXiv preprint arXiv:240509980. 2024.

29. Klein T, Walther T. Advances in Explainable Artificial Intelligence (xAI) in Finance. Elsevier; 2024.

30. Dehghan S, Amasyali MF. SupMPN: supervised multiple positives and negatives contrastive learning model for semantic textual similarity. Applied Sciences. 2022;12(19):9659.

31. Dehghan S, Amasyali MF. Selfccl: Curriculum contrastive learning by transferring self-taught knowledge for fine-tuning BERT. Applied Sciences. 2023;13(3):1913.

32. Dehghan S, Amasyali MF. A Turkish dataset and BERTurk-Contrastive model for semantic textual similarity. Journal of Information Systems and Telecommunication (JIST). 2025;1(49):24.

33. Ho VT, Ibrahim Y, Pal K, Berberich K, Weikum G. Qsearch: Answering quantity queries from text. In: International Semantic Web Conference. Springer; 2019. p. 237-57.

34. Zimina E, Nummenmaa J, Järvelin K, Peltonen J, Stefanidis K, Hyyrö H. GQA: grammatical question answering for RDF data. In: Semantic Web Evaluation Challenge. Springer; 2018. p. 82-97.

35. Zafar H, Dubey M, Lehmann J, Demidova E. IQA: Interactive query construction in semantic question answering systems. Journal of Web Semantics. 2020;64:100586.

36. Lin X, Li H, Xin H, Li Z, Chen L. KBPearl: a knowledge base population system supported by joint entity and relation linking. Proceedings of the VLDB Endowment. 2020;13(7):1035-49.

37. Sakor A, Mulang IO, Singh K, Shekarpour S, Vidal ME, Lehmann J, et al. Old is gold: linguistic driven approach for entity and relation linking of short text. In: Proceedings of the 2019 Conference of the North American Chapter of the Association for Computational Linguistics: Human Language Technologies, Volume 1 (Long and Short Papers); 2019. p. 2336-46.

38. Ni P, Okhrati R, Guan S, Chang V. Knowledge graph and deep learning-based text-to-GraphQL model for intelligent medical consultation chatbot. Information Systems Frontiers. 2024;26(1):137-56.

39. Liu Q, Feng S, Huang M. TEQA: Temporal knowledge graph enhanced question answering. Knowledge-Based Systems. 2025:113916.

40. Tapaswi N. An efficient part-of-speech tagger rule-based approach of Sanskrit language analysis. International Journal of Information Technology. 2024;16(2):901-8.

41. TG KK, Mendke S, Parihar R, Mayya S, Venkatesh S, Koolagudi SG. DBNLP: detecting bias in natural language processing system for India-centric languages. International Journal of Information Technology. 2025:1-16.

42. Formica A, Mele I, Taglino F. A template-based approach for question answering over knowledge bases. Knowledge and Information Systems. 2024;66(1):453-79.

43. Papagiannidis E, Mikalef P, Conboy K. Responsible artificial intelligence governance: A review and research framework. The Journal of Strategic Information Systems. 2025;34(2):101885.

44. De Marneffe MC, Manning CD, Nivre J, Zeman D. Universal dependencies. Computational linguistics. 2021;47(2):255-308.

45. Singh R, Konyak CY, Longkumer A. A Multi-Access Edge Computing Approach to Intelligent Tutoring Systems for Real-Time Adaptive Learning. International Journal of Information Technology. 2025;17(4):2117-28.

46. Keidar D, Zhong M, Zhang C, Shrestha YR, Paudel B. Towards automatic bias detection in knowledge graphs. arXiv preprint arXiv:210910697. 2021.

Journal: PLOS ONE
Title: Beyond Templates and BERT: Headword-Centric Parsing for Semantic Question Answering in Non-English Financial Domains
Manuscript Number: PONE-D-25-50479
Date: 10 Feb 2026

Professor Mario Graff-Guerrero
Academic Editor, PLOS One
Dear Sir,
We sincerely thank you for the painstaking review undertaken for this paper.
Thank you very much for your positive comments. They were gratifying. The constructive feedback has led to further improving the quality of the manuscript. Accordingly, we have modified the manuscript taking into consideration all the changes suggested by you.

We take it as a positive gesture that you have submitted progressive remarks about the manuscript. We have addressed the checklist.

Journal requirements:
Comment 1: "Please ensure that your manuscript meets PLOS ONE's style requirements, including those for file naming."
Response: We further updated the manuscript to meet PLOS ONE's style requirements. The manuscript now uses the provided PLOS LaTeX template.

Comment 2: "In your Methods section, please include additional information about your dataset and ensure that you have included a statement specifying whether the collection and analysis method complied with the terms and conditions for the source of the data."
Response: We have added a detailed description of the dataset, including source, collection method, preprocessing steps, and compliance with source terms. See updated Section *Result* and its Subsection *Datasets*. Part of the dataset used was manually annotated by domain experts and is considered part of the work contribution. That's why we decided to put it in Section *Results*, and not in Section *Methodology*.

Comment 3: "Please note that PLOS One has specific guidelines on code sharing for submissions in which author-generated code underpins the findings in the manuscript."

Response: We will make the code publicly available on OneDrive upon acceptance. A link will be provided in the manuscript's *Data Availability Statement*.

Comment 4: "Please update your submission to use the PLOS LaTeX template."

Response: We are thankful to the editor for appreciating our work. We have further updated the manuscript and used the PLOS LaTeX template.

Comment 5: "Data availability".

Response:

The Financial-QA dataset (2,100 German financial questions) cannot be shared publicly due to third-party licensing restrictions; however, for more informtion regarding the dataset can be made by contacting Silvio Peikert (Master of Science, Project Manager at Fraunhofer Institute for Open Communication Systems) at silvio.peikert@fokus.fraunhofer.de. The Virtuoso triple store contains proprietary financial announcements; however, a sample dataset (dbo2.json, dbo.json) is provided as Supporting Information

Reviewer 1

Comment 1: "The manuscript requires methodological clarifications: The methodology is said to rely on three theoretical pillars—Syntactic Dependency Theory, Knowledge Graph Embedding, and Human–AI Collaboration. It would be helpful if the authors could clarify how these pillars interact within the operational pipeline. While the components are described individually, the theoretical integration between them is not fully explained."

Response: We have incorporated theoretical integration and added clarifications in Section *Methodology* regarding the interaction of theoretical pillars, rule coverage, entity recognition for new companies, multi-headword conflict resolution, and similarity metrics.

Comment 2: "The system depends on predefined grammar rules in the NLTK chunker. The study would benefit from providing details on the coverage of these rules and how frequently the

parser fails due to syntactic structures that fall outside the defined grammar. Quantifying the practical impact would strengthen the discussion."

Response: We have incorporated failure analysis in the last paragraph of Subsection *Question Pre-processing*

Comment 3: "Entity recognition is restricted to company names extracted from DBPedia. It would be valuable if the authors could explain how the system handles emerging companies or entities not included in this predefined list, especially in financial domains where new firms frequently appear."

Response: We have incorporated handling emerging companies not yet in DBPedia in the last paragraph of Subsection *Question Pre-processing*

Comment 4: "Queries with multiple headwords are challenging, as noted in the study, and comparisons are made to EARL and Falcon. The paper would benefit from clarifying how INAGQA explicitly resolves conflicts arising from multiple extracted headwords."

Response: We have incorporated details of dealing with multiple headwords at the end of Subsection *Question Processing*

Comment 5: "The spelling-correction component interacts with complex or multi-word financial terms (e.g., Eigenkapitalrendite). It would be helpful if the authors could discuss how over-correction is avoided to prevent distortion of domain-specific terminology."

Response: We have incorporated spelling correction details in Subsection *Question Pre-processing*

Comment 6: "The Elasticsearch-based semantic search ranks IRIs based on similarity to headwords. The study would benefit from clarifying which similarity metric is used and how sensitive the ranking is to noisy, ambiguous, or partially extracted headwords."

Response: We have incorporated details clarifying the used similarity metric in Subsection *Workflow* starting from "*To determine the similarity between two phrases, the cosine similarity of their respective word vector is computed …*"

Comment 7: "System outputs include maps, info cards, or tables. It would be useful if the authors could explain the criteria or decision rules that determine which output format is selected for a given query."
Response: We incorporated output format and selection criteria in Subsection *Request Answer* and Subsection *Output and Curation*

Comment 8: Some newest papers are valued to refer, such as:
1. https://doi.org/10.3390/app12199659
2. https://doi.org/10.3390/app13031913
3. https://doi.org/10.61186/jist.48127.13.49.24 (Alternative link: https://jist.ir/en/Article/48127)
Response: We added all suggested references in Section Related Work

Specific Points Addressed:

- Theoretical integration added at the beginning of Section *Methodology*.
- Grammar rule coverage detailed in Subsection *Workflow*
- Entity recognition for new companies addressed via Wikidata fallback (Subsection *Suggestion*).
- Multi-headword conflict resolution via similarity ranking (Subsection *SPARQL Mapping*).
- Spelling correction details added (Subsection *Question Pre-processing*).
- Similarity metric specified as cosine similarity with BERT embeddings (Subsection *SPARQL Mapping*).
- Output format selection criteria added in Subsections *Request Answer* and *Output and Curation*
- Suggested references have been reviewed and cited where relevant.

Reviewer 2

Comment 1: "However, the paper is lengthy and can be shorten as a concise version. For example, the workflow section is excessively detailed, including explanations of well-established concepts such as POS-tagging that are common knowledge in the NLP community. The paper would be more accessible if streamlined to focus on the novel contributions rather than providing textbook-level explanations of standard techniques."

Response: We thank the reviewer for the valuable suggestion. We have significantly streamlined the manuscript, particularly in the *Methodology* section, by removing basic NLP explanations (e.g., detailed descriptions of POS-tagging and syntactic parsing). The revised paper now focuses on our novel contributions: the headword-centric parsing algorithm, the hybrid rule- and embedding-based architecture, and the system's practical integration.

Comment 2: "While the authors present a carefully designed workflow, the rationale behind key methodological choices is not well explained. For the first step, they use syntactic parsing to do pattern match, is it scalable or how is the coverage of this pattern detection? Why not any DL-model based solutions? In addition, in order to generate SPARQL query, they used Spacy's standard NER, why not choose any other deep learning methods. Can you add a dedicated subsection justifying each major architectural choice with empirical evidence or theoretical reasoning?"

Response: We have added a new dedicated subsection in Section *Methodology* subsection *Rationale for Architectural Choices*. This subsection explicitly addresses the reviewer's questions:

Scalability and Coverage of Pattern Matching: We clarify that the system employs 46 handcrafted grammar rules, which achieved 92% coverage on our test set of 2,100 questions. Failure cases and error analysis are discussed in Subsection *Question Pre-processing*.

Choice of Rule-Based Parsing over Deep Learning (DL): We justify this choice based on: (1) Interpretability and control for decomposing German compound nouns, (2) Data efficiency, as large annotated German financial QA datasets are scarce, and (3) Lower operational latency and cost compared to querying large LLM APIs, which is crucial for enterprise deployment.

Choice of SpaCy NER: We explain that SpaCy's de_core_news_lg model offers robust, fast entity recognition for German. As our core financial entity list (companies) is stable, we

augmented it with a custom EntityRuler for domain terms, achieving high precision without the need for training a custom DL model.

Comment 3: “The scalability and generalizability of the approach remain uncertain. The system relies on handwritten grammar rules for pattern matching, but the paper does not clarify: (1) how many rules were created for the 2,100 test questions, (2) what coverage can be expected for unseen question variations, and (3) how much expert effort is required to extend the system to new financial concepts or question types. This raises concerns about the practical scalability of the rule-based approach.”

Response: We have provided the requested clarifications:

Number of Rules: As stated above, 46 handcrafted grammar rules were developed (Subsection *Rationale for Architectural Choices*).

Coverage for Unseen Variations: These rules covered 92% of the 2,100 test questions. We discuss the nature of the 8% failure cases (primarily highly elliptical or metaphoric questions) and our fallback strategy using embedding-based similarity ranking in Subsection *Question Pre-processing*

Expert Effort for Extension: The modular design of the rule system allows incremental extension. Adding a new question pattern or financial concept typically requires 1-2 new rules, which can be authored by a domain expert in collaboration with a developer. This process and its manageability are discussed in Subsection *Limitations and Future Work*.

Comment 4: “With recent advances in LLMs, many sub-tasks can now be automated through in-context learning. However, the paper lacks comparison with current SOTA approaches such as GPT-4, Claude, or fine-tuned multilingual LLMs. It is more of a comparison of rule-based methods v.s. automated solutions.”

Response: We agree that a comparison with modern LLMs is essential. We have added a new part in Section *Discussion* Subsection *Comparison with Large Language Models*. This section reports a comparative evaluation of GPT-4 (using few-shot prompting) on a subset of our Financial-QA dataset.

discusses the results: While GPT-4 performs well on factual queries, INAGQA maintains a significant advantage (15% higher F1-score) on ambiguous queries and complex German compound nouns.
provides a reasoned discussion on the trade-offs: LLMs offer generality but at higher cost, latency, and with less interpretability and control for specific, sensitive financial domains and highlights the continued relevance of our specialized, hybrid approach.

Reviewer 3
Comment I-III (Weaknesses): "Concerns regarding data availability, experimental setup clarity, baseline configuration, absence of ablation study and LLM comparison, and claims vs. evidence on cross-domain applicability."
Response to Weaknesses: We thank the reviewer for these critical points, which have significantly strengthened the manuscript.
Data Availability: We have the manuscript and added section *Data Availability Statement* in full compliance with PLOS ONE policy. The proprietary Financial-QA dataset (2,100 questions) will be made available upon request for research purposes. A sample dataset (dbo.json) is provided as Supporting Information.
Dataset details, including source, collection method, preprocessing, and annotation guidelines, are now provided in section *Results* subsection *Datasets*. We also report an inter-annotator agreement (IAA) score (Cohen's $\kappa = 0.85$) for the annotated questions.
Experimental Setup & Baselines: We clarify that all baseline systems (Falcon 2.0, EARL, BERT-KGQA) were retrained/fine-tuned on our German financial dataset to ensure a fair comparison. Training details (data splits, hyperparameters) are provided in section *Results* subsection *Performance Comparison*.
The specific embedding model used for similarity ranking in INAGQA is bert-base-german-cased, as now specified in section *Methodology* subsection *SPARQL Mapping*.
Ablation Study: We have added a new Section *Results* subsection *Ablation Study*. This study isolates the contribution of key components (pattern matching, headword extraction, similarity ranking), clearly demonstrating that the full hybrid pipeline delivers the best performance.

LLM Comparison: ~~As detailed in the response to Reviewer #2 (Comment 4),~~ we have added a comprehensive comparison with GPT-4 in Section *Discussion* Subsection *Comparison with Large Language Models*.

Cross-Domain Applicability: We have clarified the results in Table 6. The performance drop in healthcare/legal domains occurred when using financial-domain rules without modification. We note that adapting the system requires developing domain-specific rules, which is feasible given the modular architecture. This discussion on generalizability is now in Section *Conclusion and Further Work* Subsection *Methodological Innovation*.

Comment IV (Questions to Authors):

Q1. Dataset specification and experimental setup:

Datasets: Tables 4, 5, and 6 use our Financial-QA dataset. The Virtuoso triple store (14k announcements) was used for the case study in Section *Results* Subsection *Datasets*.

Train/Test Split: The dataset was split 80/20 (1,680 training / 420 test questions) for development and final evaluation. All reported results (including baselines) are on the held-out test set.

Q2. Baseline configuration: All baselines were fine-tuned for German using our training split. We used the authors' original code where available and adapted training pipelines for the German financial domain. Specifics are in Section *Results* Subsection *Experimental Setup*.

Embedding models: INAGQA uses bert-base-german-cased. BERT-KGQA used the same for fairness. Falcon and EARL used their standard multilingual embeddings, as per their design.

Q3. Cross-domain evaluation: Healthcare/legal test questions were sourced from public German QA benchmarks and curated with domain experts. For Table 6, we applied the Financial-QA rules without modification to test "out-of-the-box" performance, hence the noted drop. Successful adaptation, as discussed, requires adding domain-specific rules.

Comment V (Writing style and organizational suggestions):

Redundant Content: We have consolidated the manuscript, removing duplicate explanations of entity recognition and SPARQL generation (now primarily in Section *Methodology*).

Undefined Terms: We now define **Corporate Smart Insights** (our overarching analytics framework) and **OMG API** (the gateway for knowledge base queries) upon their first use in Section *Introduction* and Section *Architecture*.

Comment VI (Typographical errors):

Table 7 Legend: Added a clear legend: "✓ indicates correct answer, o indicates failure or incorrect answer."

Incorrect Reference: Corrected the reference in Section *Discussion* from *Table 2* to *Table 7*.

Reviewer 4

Comment 1.1 & 1.2 (Data Availability & Annotation):

Response: We have fully addressed these concerns.

The *Data Availability Statement* (Section *Conclusion and Further Work*) now clearly states the conditions for accessing the proprietary Financial-QA dataset and provides a sample (dbo.json). In Section *Results* Subsection *Datasets*, we detail the annotation process. The 2,100 questions were annotated by two domain experts. We report an inter-annotator agreement (IAA) score of Cohen's $\kappa = 0.85$, indicating substantial agreement. Discrepancies were resolved through discussion.

Comment 2.1 & 2.2 (Evaluation - Baseline Fairness & Cross-Validation):

Response: We thank the reviewer for highlighting the need for rigorous evaluation.

Baseline Fairness: We confirm that all baseline systems (Falcon 2.0, EARL, BERT-KGQA) were fine-tuned on the same German financial training data (1,680 questions) as INAGQA. Hyperparameter tuning details and training convergence criteria are provided in Section *Results* Subsection *Performance Comparision*.

Cross-Validation: To provide a more robust performance estimate, we have conducted 5-fold cross-validation on the Financial-QA dataset. The results (average F1-score of 0.89 ± 0.02) are presented in a new Table 5. The headline F1-score of 0.91 in the abstract and results refers to the performance on the final, held-out test set, consistent with the cross-validation average.

CONCLUDING REMARKS

We thank the reviewers and editor for their valuable feedback, which has significantly improved the manuscript. Since all the changes that you requested have been incorporated, we sincerely hope that the manuscript is in its final publishable form.

With Gratitude,
The Authors
