# Supplementary material for: Beyond Templates and BERT: Headword-centric parsing for semantic question answering in non-english financial domains
Source: PLoS One. 2026 May 4;21(5):e0347261. doi: 10.1371/journal.pone.0347261 (PMC13138744; doi:10.1371/journal.pone.0347261)
Supplement: Attachment — Submitted filename: Response to Reviewers Comment revision2.pdf [file pone.0347261.s007.pdf]

Journal: PLOS ONE

Title: Beyond Templates and BERT: Headword-Centric Parsing for Semantic Question Answering in Non-English Financial Domains

Manuscript Number: PONE-D-25-50479

Date: 12 March 2026

Professor Mario Graff-Guerrero

Academic Editor, PLOS One

Dear Sir,

We sincerely thank the editor and reviewers for their careful reading of the manuscript and for their constructive feedback. We are grateful that the manuscript has progressed to this final stage. We appreciate that two reviewers now recommend publication, and we have carefully considered the remaining presentation-related comments. Below, we respond point by point, relying on clarifications already reflected in the revised manuscript.

With Gratitude,

The Authors

Journal Requirements

Comment 1: If the reviewer comments include a recommendation to cite specific previously published works, please review and evaluate these publications to determine whether they are relevant and should be cited.

Response:

In the present and previous review rounds, there is no additional citation that was requested by the reviewers

Comment 2: Please review your reference list to ensure that it is complete and correct. If you have cited papers that have been retracted.

Response:

We carefully reviewed the reference list for completeness, correctness, and retraction status. We confirm that none of the references cited in the manuscript is retracted. Therefore, no retracted references were removed or replaced.

EDITORIAL COMMENTS

Comment 1. Define the term "Headword" in the Introduction

Response:
We have added a clear definition of the term Headword, including an example, at the end of the Introduction. This definition clarifies the linguistic role of headwords in intent classification and aligns with the manuscript's focus. Aloso The manuscript already explains the role of headwords in the processing pipeline and provides illustrative examples in the Methodology section. In particular, the manuscript explains that in a query such as "What is the X of Siemens?", the term X functions as the headword for intent classification, and in more complex examples such as "Where was Siemens founded?", multiple headwords are extracted and disambiguated through similarity ranking. The Introduction also motivates this by discussing question ambiguity, such as the distinction between Where was Siemens founded? and When was Siemens founded?

Comment 2. Define all acronyms upon first use (IS, RDF, IMG, AI, etc.)

Response:
We reviewed the manuscript and ensured that each acronym is defined at first appearance.

Comment 3. Two DBPedia references (Refs 4 and 13)

Response:
We confirmed that both DBpedia citations refer to two different foundational publications (2007 and later) , where the references associated with DBpedia and related prior work to ensure that each citation serves a distinct purpose in the manuscript. Reference [13] is the foundational DBpedia paper, whereas Reference [4] is a more recent DBpedia-based application in another language context; they are therefore not duplicate references, but serve different roles in the literature review. Both are retained intentionally due to their separate relevance.

Comment 4. The sections Headword Extraction Techniques, Financial Domain Applications, and Proposed Work are overused. At first, these sections seem more like slides in a presentation than a description of a journal paper. Some of the items could be described in more detail.

Response:

We appreciate this stylistic suggestion. The intention of these short subsections was to provide a compact synthesis of the related-work landscape before moving into the fuller technical discussion that follows in the Review, Architecture, and Methodology sections. In the current manuscript, these subsections function as structured signposts that summarize the problem space, while the detailed operational treatment of headword extraction, relation linking, output generation, and system workflow is presented later in the paper.

Comment 5. The information presented in Table 1 is important because it summarizes your approach. Unfortunately, the table is barely described in the manuscript, so it took me a while to understand.

Response :

Thank you for highlighting this. Table 1 was intended as a concise summary of how INAGQA positions itself relative to template-based, neural, and hybrid approaches. Its logic is introduced in the Related Work section and then elaborated through the subsequent discussion of AskNow, EARL, Falcon, and the later performance and workflow sections. In that sense, Table 1 is meant as a high-level synthesis, where Table 1 is already cited and explained clearly in the Related Work section and again in the "Proposed Work" section. Therefore, no further modification was necessary.

Reviewer #1

Comment: All comments have been addressed.

Response:

We sincerely thank the reviewer for the positive assessment and for the constructive feedback throughout the review process.

Reviewer #2

Comment 1. The paper still needs to be more concise and well-structured to be accepted.

Response:

We thank the reviewer for this constructive recommendation. The revised manuscript already reflects substantial consolidation of the presentation. In particular, the first-round revision streamlined the Methodology section by removing basic textbook-style explanations and focusing more directly on the manuscript's novel contributions, namely headword-centric parsing, hybrid rule-plus-embedding disambiguation, and practical knowledge-graph integration.

Comment 2 . Move "Rationale for Architectural Choices" earlier... integrate it into the start of the Methodology.

Response:

We appreciate this suggestion. The manuscript already includes a dedicated subsection, Rationale for Architectural Choices, within the Methodology section. This subsection explicitly explains the reasons for selecting rule-based parsing, SpaCy NER, and the modular grammar-rule design, including interpretability for German compound nouns, data efficiency, and lower latency/cost for enterprise deployment. This rationale is intended to frame the subsequent implementation details.

Comment 3. In Related Work section: Instead of long paragraphs describing every previous system, use a comparison table to show how INAGQA differs from systems like AskNow, EARL, and Falcon.

Response:

We agree with the value of comparative synthesis, and the current manuscript already provides this in tabular form. Specifically, Table 1 gives a high-level comparison of approach families, while Table 2 provides a direct comparison of AskNow, EARL, and Falcon in terms of approach, implementation, evaluation, and advantages/disadvantages. These tables were included precisely to make the positioning of INAGQA clearer. We also We added a brief pointer sentence to the end Related Work section to highlight the table (A comparative summary of the main systems discussed in this section is presented in Table 2).

Comment4. You can significantly clean up the paper by "offloading" technical data. For example, rather than detailing all 46 handcrafted grammar rules in the text, provide a high-level summary of their 92% coverage and move the full list to an Appendix.

Response:

Thank you for this helpful recommendation. In the current revised manuscript, we already summarize the grammar-rule component at a high level rather than enumerating all rules exhaustively. The manuscript reports that the system uses 46 handcrafted grammar rules and that these cover 92% of the 2,100 Financial-QA questions, while the workflow section presents only representative rule patterns needed to understand the approach. The aim was to preserve readability while still providing sufficient methodological transparency and reproducibility.

Reviewer #4

Comment: The author has successfully addressed all comments; therefore, the paper is now ready for publication.

Response: We sincerely thank the reviewer for the positive assessment and for the constructive feedback throughout the review process.
