# Supplementary material for: Beyond Templates and BERT: Headword-centric parsing for semantic question answering in non-english financial domains
Source: PLoS One. 2026 May 4;21(5):e0347261. doi: 10.1371/journal.pone.0347261 (PMC13138744; doi:10.1371/journal.pone.0347261)
Supplement: Attachment — Submitted filename: Response to Reviewers Comments.docx [file pone.0347261.s005.docx]

Journal: PLOS ONE

Title: Beyond Templates and BERT: Headword-Centric Parsing for Semantic Question Answering in Non-English Financial Domains

Manuscript Number: PONE-D-25-50479

Date: 10 Feb 2026

Professor Mario Graff-Guerrero

Academic Editor, PLOS One

Dear Sir,

We sincerely thank you for the painstaking review undertaken for this paper.

Thank you very much for your positive comments. They were gratifying. The constructive feedback has led to further improving the quality of the manuscript. Accordingly, we have modified the manuscript taking into consideration all the changes suggested by you.

We take it as a positive gesture that you have submitted progressive remarks about the manuscript. We have addressed the checklist.

**Journal requirements:**

**Comment 1:** “Please ensure that your manuscript meets PLOS ONE's style requirements, including those for file naming.”

**Response:** We further updated the manuscript to meet PLOS ONE's style requirements. The manuscript now uses the provided PLOS LaTeX template.

**Comment 2:** “In your Methods section, please include additional information about your dataset and ensure that you have included a statement specifying whether the collection and analysis method complied with the terms and conditions for the source of the data.”

**Response:** We have added a detailed description of the dataset, including source, collection method, preprocessing steps, and compliance with source terms. See updated Section *Result* and its Subsection *Datasets*. Part of the dataset used was manually annotated by domain experts and is considered part of the work contribution. That's why we decided to put it in Section *Results*, and not in Section *Methodology*.

**Comment 3:** “Please note that PLOS One has specific guidelines on code sharing for submissions in which author-generated code underpins the findings in the manuscript.”

**Response:** We will make the code publicly available on OneDrive upon acceptance. A link will be provided in the manuscript’s *Data Availability Statement*.

**Comment 4:** “Please update your submission to use the PLOS LaTeX template.”

**Response:** We are thankful to the editor for appreciating our work. We have further updated the manuscript and used the PLOS LaTeX template.

**Comment 5:** “Data availability”.

**Response:**

The Financial-QA dataset (2,100 German financial questions) cannot be shared publicly due to third-party licensing restrictions; however, for more informtion regarding the dataset can be made by contacting Silvio Peikert (Master of Science, Project Manager at Fraunhofer Institute for Open Communication Systems) at silvio.peikert@fokus.fraunhofer.de. The Virtuoso triple store contains proprietary financial announcements; however, a sample dataset (dbo2.json, dbo.json) is provided as Supporting Information

**Reviewer 1**

**Comment 1:** “The manuscript requires methodological clarifications: The methodology is said to rely on three theoretical pillars—Syntactic Dependency Theory, Knowledge Graph Embedding, and Human–AI Collaboration. It would be helpful if the authors could clarify how these pillars interact within the operational pipeline. While the components are described individually, the theoretical integration between them is not fully explained.”
**Response:** We have incorporated theoretical integration and added clarifications in Section *Methodology* regarding the interaction of theoretical pillars, rule coverage, entity recognition for new companies, multi-headword conflict resolution, and similarity metrics.

**Comment 2:** “The system depends on predefined grammar rules in the NLTK chunker. The study would benefit from providing details on the coverage of these rules and how frequently the parser fails due to syntactic structures that fall outside the defined grammar. Quantifying the practical impact would strengthen the discussion.”

**Response:** We have incorporated failure analysis in the last paragraph of Subsection *Question Pre-processing*

**Comment 3:** “Entity recognition is restricted to company names extracted from DBPedia. It would be valuable if the authors could explain how the system handles emerging companies or entities not included in this predefined list, especially in financial domains where new firms frequently appear.”

**Response:** We have incorporated handling emerging companies not yet in DBPedia in the last paragraph of Subsection *Question Pre-processing*

**Comment 4:** “Queries with multiple headwords are challenging, as noted in the study, and comparisons are made to EARL and Falcon. The paper would benefit from clarifying how INAGQA explicitly resolves conflicts arising from multiple extracted headwords.”

**Response:** We have incorporated details of dealing with multiple headwords at the end of Subsection *Question Processing*

**Comment 5:** “The spelling-correction component interacts with complex or multi-word financial terms (e.g., Eigenkapitalrendite). It would be helpful if the authors could discuss how over-correction is avoided to prevent distortion of domain-specific terminology.”

**Response:** We have incorporated spelling correction details in Subsection *Question Pre-processing*

**Comment 6:** “The Elasticsearch-based semantic search ranks IRIs based on similarity to headwords. The study would benefit from clarifying which similarity metric is used and how sensitive the ranking is to noisy, ambiguous, or partially extracted headwords.”

**Response:** We have incorporated details clarifying the used similarity metric in Subsection *Workflow* starting from “*To determine the similarity between two phrases, the cosine similarity of their respective word vector is computed* …”

**Comment 7:** “System outputs include maps, info cards, or tables. It would be useful if the authors could explain the criteria or decision rules that determine which output format is selected for a given query.”

**Response:** We incorporated output format and selection criteria in Subsection *Request Answer* and Subsection *Output and Curation*

**Comment 8:** Some newest papers are valued to refer, such as:

1. https://doi.org/10.3390/app12199659

2. https://doi.org/10.3390/app13031913

3. https://doi.org/10.61186/jist.48127.13.49.24 (Alternative link: https://jist.ir/en/Article/48127)

**Response:**  We added all suggested references in Section Related Work

**Specific Points Addressed:**

- Theoretical integration added at the beginning of Section *Methodology*.
- Grammar rule coverage detailed in Subsection *Workflow*
- Entity recognition for new companies addressed via Wikidata fallback (Subsection *Suggestion*).
- Multi-headword conflict resolution via similarity ranking (Subsection *SPARQL Mapping*).
- Spelling correction details added (Subsection *Question Pre-processing*).
- Similarity metric specified as cosine similarity with BERT embeddings (Subsection *SPARQL Mapping*).
- Output format selection criteria added in Subsections *Request Answer* and *Output and Curation*
- Suggested references have been reviewed and cited where relevant.

**Reviewer 2**

**Comment 1:** “However, the paper is lengthy and can be shorten as a concise version. For example, the workflow section is excessively detailed, including explanations of well-established concepts such as POS-tagging that are common knowledge in the NLP community. The paper would be more accessible if streamlined to focus on the novel contributions rather than providing textbook-level explanations of standard techniques.”

**Response:** We thank the reviewer for the valuable suggestion. We have significantly streamlined the manuscript, particularly in the *Methodology* section, by removing basic NLP explanations (e.g., detailed descriptions of POS-tagging and syntactic parsing). The revised paper now focuses on our novel contributions: the headword-centric parsing algorithm, the hybrid rule- and embedding-based architecture, and the system's practical integration.

**Comment 2:** “While the authors present a carefully designed workflow, the rationale behind key methodological choices is not well explained. For the first step, they use syntactic parsing to do pattern match, is it scalable or how is the coverage of this pattern detection? Why not any DL-model based solutions? In addition, in order to generate SPARQL query, they used Spacy’s standard NER, why not choose any other deep learning methods. Can you add a dedicated subsection justifying each major architectural choice with empirical evidence or theoretical reasoning?”

**Response:** We have added a new dedicated subsection in Section *Methodology* subsection ***Rationale for Architectural Choices***. This subsection explicitly addresses the reviewer's questions:

**Scalability and Coverage of Pattern Matching:** We clarify that the system employs **46 handcrafted grammar rules**, which achieved **92% coverage** on our test set of 2,100 questions. Failure cases and error analysis are discussed in Subsection *Question Pre-processing*.

**Choice of Rule-Based Parsing over Deep Learning (DL):** We justify this choice based on: (1) **Interpretability and control** for decomposing German compound nouns, (2) **Data efficiency**, as large annotated German financial QA datasets are scarce, and (3) **Lower operational latency and cost** compared to querying large LLM APIs, which is crucial for enterprise deployment.

**Choice of SpaCy NER:** We explain that SpaCy's de_core_news_lg model offers robust, fast entity recognition for German. As our core financial entity list (companies) is stable, we augmented it with a custom EntityRuler for domain terms, achieving high precision without the need for training a custom DL model.

**Comment 3:** “The scalability and generalizability of the approach remain uncertain. The system relies on handwritten grammar rules for pattern matching, but the paper does not clarify: (1) how many rules were created for the 2,100 test questions, (2) what coverage can be expected for unseen question variations, and (3) how much expert effort is required to extend the system to new financial concepts or question types. This raises concerns about the practical scalability of the rule-based approach.”

**Response:** We have provided the requested clarifications:

**Number of Rules:** As stated above, **46 handcrafted grammar rules** were developed (Subsection *Rationale for Architectural Choices*).

**Coverage for Unseen Variations:** These rules covered **92%** of the 2,100 test questions. We discuss the nature of the 8% failure cases (primarily highly elliptical or metaphoric questions) and our fallback strategy using embedding-based similarity ranking in Subsection *Question Pre-processing*

**Expert Effort for Extension:** The modular design of the rule system allows incremental extension. Adding a new question pattern or financial concept typically requires 1-2 new rules, which can be authored by a domain expert in collaboration with a developer. This process and its manageability are discussed in Subsection *Limitations and Future Work*.

**Comment 4:** “With recent advances in LLMs, many sub-tasks can now be automated through in-context learning. However, the paper lacks comparison with current SOTA approaches such as GPT-4, Claude, or fine-tuned multilingual LLMs. It is more of a comparison of rule-based methods v.s. automated solutions.”

**Response:** We agree that a comparison with modern LLMs is essential. We have added a new part in Section *Discussion* Subsection ***Comparison with Large Language Models***. This section

reports a comparative evaluation of **GPT-4** (using few-shot prompting) on a subset of our Financial-QA dataset.

discusses the results: While GPT-4 performs well on factual queries, INAGQA maintains a significant advantage (**15% higher F1-score**) on ambiguous queries and complex German compound nouns.

provides a reasoned discussion on the trade-offs: LLMs offer generality but at higher cost, latency, and with less interpretability and control for specific, sensitive financial domains and highlights the continued relevance of our specialized, hybrid approach.

**Reviewer 3**

**Comment I-III (Weaknesses):** “Concerns regarding data availability, experimental setup clarity, baseline configuration, absence of ablation study and LLM comparison, and claims vs. evidence on cross-domain applicability.”

**Response to Weaknesses:** We thank the reviewer for these critical points, which have significantly strengthened the manuscript.

**Data Availability:** We have the manuscript and added section ***Data Availability Statement*** in full compliance with PLOS ONE policy. The proprietary **Financial-QA dataset** (2,100 questions) will be made available upon request for research purposes. A **sample dataset** (dbo.json) is provided as Supporting Information.

**Dataset details**, including source, collection method, preprocessing, and annotation guidelines, are now provided in section *Results* subsection *Datasets*. We also report an **inter-annotator agreement (IAA) score (Cohen’s κ = 0.85)** for the annotated questions.

**Experimental Setup & Baselines:** We clarify that all baseline systems (**Falcon 2.0, EARL, BERT-KGQA**) were **retrained/fine-tuned on our German financial dataset** to ensure a fair comparison. Training details (data splits, hyperparameters) are provided in section *Results* subsection *Performance Comparison*.

The specific **embedding model** used for similarity ranking in INAGQA is bert-base-german-cased, as now specified in section *Methodology* subsection *SPARQL Mapping*.

**Ablation Study:** We have added a new **Section *Results* subsection *Ablation Study***. This study isolates the contribution of key components (pattern matching, headword extraction, similarity ranking), clearly demonstrating that the full hybrid pipeline delivers the best performance.

**LLM Comparison:** ~~As detailed in the response to Reviewer #2 (Comment 4),~~ we have added a comprehensive comparison with **GPT-4** in Section *Discussion* Subsection *Comparison with Large Language Models*.

**Cross-Domain Applicability:** We have clarified the results in Table 6. The performance drop in healthcare/legal domains occurred when using **financial-domain rules without modification**. We note that adapting the system requires developing **domain-specific rules**, which is feasible given the modular architecture. This discussion on generalizability is now in Section *Conclusion and Further Work* Subsection *Methodological Innovation*.

**Comment IV (Questions to Authors):**

**Q1. Dataset specification and experimental setup:**

**Datasets:** Tables 4, 5, and 6 use our **Financial-QA dataset**. The Virtuoso triple store (14k announcements) was used for the case study in Section *Results* Subsection *Datasets*.

**Train/Test Split:** The dataset was split **80/20 (1,680 training / 420 test questions)** for development and final evaluation. All reported results (including baselines) are on the **held-out test set**.

**Q2. Baseline configuration:** All baselines were **fine-tuned for German** using our training split. We used the authors' original code where available and adapted training pipelines for the German financial domain. Specifics are in Section *Results* Subsection *Experimental Setup*.

**Embedding models:** INAGQA uses bert-base-german-cased. BERT-KGQA used the same for fairness. Falcon and EARL used their standard multilingual embeddings, as per their design.

**Q3. Cross-domain evaluation:** Healthcare/legal test questions were sourced from **public German QA benchmarks and curated with domain experts**. For Table 6, we applied the **Financial-QA rules without modification** to test "out-of-the-box" performance, hence the noted drop. Successful adaptation, as discussed, requires adding domain-specific rules.

**Comment V (Writing style and organizational suggestions):**

**Redundant Content:** We have consolidated the manuscript, removing duplicate explanations of entity recognition and SPARQL generation (now primarily in Section *Methodology*).

**Undefined Terms:** We now define **Corporate Smart Insights** (our overarching analytics framework) and **OMG API** (the gateway for knowledge base queries) upon their first use in Section *Introduction* and Section *Architecture*.

**Comment VI (Typographical errors):**

**Table 7 Legend:** Added a clear legend: "✓ indicates correct answer, o indicates failure or incorrect answer."

**Incorrect Reference:** Corrected the reference in Section *Discussion* from *Table 2* to ***Table 7***.

**Reviewer 4**

**Comment 1.1 & 1.2 (Data Availability & Annotation):**
**Response:** We have fully addressed these concerns.

The ***Data Availability Statement*** (Section *Conclusion and Further Work*) now clearly states the conditions for accessing the proprietary Financial-QA dataset and provides a sample (dbo.json).

In Section *Results* Subsection *Datasets*, we detail the **annotation process**. The 2,100 questions were annotated by **two domain experts**. We report an **inter-annotator agreement (IAA) score of Cohen’s κ = 0.85**, indicating substantial agreement. Discrepancies were resolved through discussion.

**Comment 2.1 & 2.2 (Evaluation - Baseline Fairness & Cross-Validation):**
**Response:** We thank the reviewer for highlighting the need for rigorous evaluation.

**Baseline Fairness:** We confirm that all baseline systems (**Falcon 2.0, EARL, BERT-KGQA**) were **fine-tuned on the same German financial training data (1,680 questions)** as INAGQA. Hyperparameter tuning details and training convergence criteria are provided in Section *Results* Subsection *Performance Comparision*.

**Cross-Validation:** To provide a more robust performance estimate, we have conducted **5-fold cross-validation** on the Financial-QA dataset. The results (average F1-score of **0.89 ± 0.02**) are presented in a new **Table 5**. The headline F1-score of 0.91 in the abstract and results refers to the performance on the final, held-out test set, consistent with the cross-validation average.

**CONCLUDING REMARKS**

We thank the reviewers and editor for their valuable feedback, which has significantly improved the manuscript. Since all the changes that you requested have been incorporated, we sincerely hope that the manuscript is in its final publishable form.

With Gratitude,

The Authors
